# Entinostat as a combinatorial therapeutic for rhabdomyosarcoma

**DOI:** 10.1038/s41598-024-66545-5

**Published:** 2024-08-15

**Authors:** Shefali Chauhan, Emily Lian, Iman Habib, Qianqian Liu, Nicole M. Anders, Megan M. Bugg, Noah C. Federman, Joel M. Reid, Clinton F. Stewart, Tristan Cates, Joel E. Michalek, Charles Keller

**Affiliations:** 1 Children’s Cancer Therapy Development Institute, 9025 NE Von Neumann Drive Ste 110, Hillsboro, OR 97006 USA; 2 Champions Oncology, Rockville, MD 20850 USA; 3 Department of Epidemiology and Biostatistics, University of Texas Health Science Center San Antonio, San Antonio, TX 78229 USA; 4 Department of Oncology, School of Medicine, Johns Hopkins University, Baltimore, MD 21231 USA; 5 The Sidney Kimmel Comprehensive Cancer Center at Johns Hopkins University, Baltimore, MD 21231 USA; 6 Jonsson Comprehensive Cancer Center, University of California Los Ángeles, Los Angeles, CA USA; 7 Department of Pediatrics, University of California, Los Angeles, Los Angeles, CA USA; 8 Mayo Clinic Comprehensive Cancer Center, Rochester, MN 55905 USA; 9 Pharmacy and Pharmaceutical Sciences, St. Jude Children’s Research Hospital, Memphis, TN 38105 USA; 10 Present Address: Takeda Pharmaceutical Company, San Diego, CA 92121 USA

**Keywords:** Entinostat, Mocetinostat, Rhabdomyosarcoma, Alveolar rhabdomyosarcoma, Embryonal rhabdomyosarcoma, HDAC inhibitor, Biological models, Biochemistry, Cell biology, Biologics, Drug safety, Pharmaceutics, Pharmacology, Target identification, Target validation, Cancer, Cancer models, Cancer therapy, Proteins, Screening, Small molecules

## Abstract

Rhabdomyosarcoma (RMS) is the most common childhood soft tissue sarcoma. For the alveolar subtype (ARMS), the presence of the *PAX3::FOXO1* fusion gene and/or metastases are strong predictors of poor outcome. Metastatic *PAX3::FOXO1*^+^ ARMS often responds to chemotherapies initially, only to subsequently relapse and become resistant with most patients failing to survive beyond 8 years post-diagnosis. No curative intent phase II or phase III clinical trial has been available for patients in the past 10 years (ARST0921). Thus, metastatic ARMS represents a significantly unmet clinical need. Chemotherapy resistance in ARMS has previously been attributed to PAX3::FOXO1-mediated cell cycle checkpoint adaptation, which is mediated by an HDAC3-SMARCA4-*miR-27a*-PAX3::FOXO1 circuit that can be disrupted by HDAC3 inhibition. In this study, we investigated the therapeutic efficacy of combining the epigenetic regulator entinostat, a Class I Histone Deacetylase (HDAC1-3) inhibitor, with RMS-specific chemotherapies in patient derived xenograft (PDX) models of RMS. We identified single agent, additive or synergistic relationships between relapse-specific chemotherapies and clinically relevant drug exposures of entinostat in three *PAX3::FOXO1*^+^ ARMS mouse models. This preclinical data provides further rationale for clinical investigation of entinostat, already known to be well tolerated in a pediatric phase I clinical trial (ADVL1513).

## Introduction

Rhabdomyosarcoma (RMS) accounts for 50% of all pediatric soft tissue sarcomas and is comprised of four major subtypes: embryonal (60%), alveolar (20%), pleomorphic/anaplastic (10%) and spindle/sclerosing (10%)^1^. Alveolar rhabdomyosarcoma (ARMS) differs from embryonal rhabdomyosarcoma (ERMS) not only in morphology, as their names imply, but also in mutational landscape, as ERMS often features gain-of-function oncogenes (commonly RAS pathway mutations) whereas ARMS features translocation-mediated *PAX* fusion oncogenes^2^. Most commonly the ARMS fusions are *PAX3::FOXO1* or *PAX7::FOXO1*. These fusions are thought to mediate cell cycle checkpoint adaptation^3–5^. Correspondingly, fusion positive (*PAX3::FOXO1*) tumors have been shown to have higher rates of chemotherapy resistance, metastasis, and recurrence with a dismal prognosis^6–8^.

**Figure 1:**
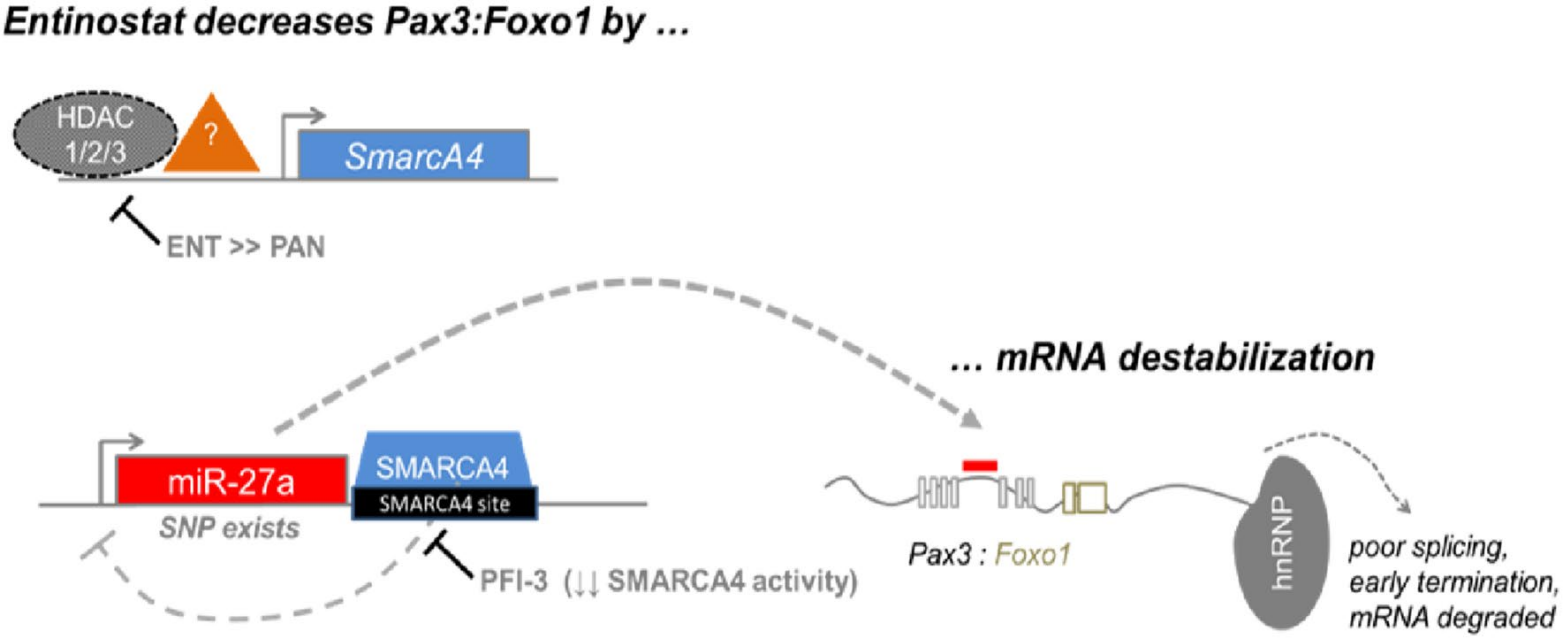
Diagrammatic representation of the HDAC3-SMARCA4-*miR-27a*-PAX3::FOXO1 circuit, which can be disrupted by entinostat or SMARCA4 bromodomain activity inhibitor, PFI-3. Adapted from 3.

Current frontline chemotherapeutic standard of care treatment (vincristine, actinomycin, and cyclophosphamide) for fusion positive and negative RMS can be curative in the early stages of the disease^9^, whereas a number of other chemotherapies have been investigated for refractory/relapsed disease including irinotecan, vinorelbine, cyclophosphamide, doxorubicin and topotecan. On the other hand, metastatic fusion positive RMS has a 5-year disease free survival rate of only 8% and a high incidence of chemotherapy resistance^1^. Recent publications have highlighted the epigenetic mechanism of cell cycle checkpoint adaptation as a possible cause of resistance^3,4,10^. SMARCA4 is upregulated preferentially over SMARCA2 in fusion positive RMS, with both SMARCA4 and SMARCA2 being ATPase components of the SWI/SNF complex that alters chromatin for the regulation of genes and onset of differentiation. Bharathy and colleagues found that the histone deacetylase 3 (HDAC3) regulates *SMARCA4,* which in turn inhibits *miR-27a*, resulting in sustained expression of the *PAX3::FOXO1* fusion oncogene. Inhibition of this pathway using entinostat decreases SMARCA4 and allows for the expression of *miR-27a*, resulting in chemotherapy sensitization in a time frame of 72 h (Fig. 1)^3^ and as a separate process from longer-term chromatin remodeling^11–14^. Separately, Bharathy and colleagues also observed single agent activity of entinostat for 50% of fusion negative RMS models, keeping with related reports of HDAC3 inhibition in ERMS^15,16^.

We sought to expand on these studies by newly investigating the therapeutic efficacy of entinostat in combination with a series of rhabdomyosarcoma relapse-related chemotherapies in both ARMS and ERMS in vivo which could support the development of a human subject clinical trial in refractory and relapsed rhabdomyosarcoma. In pediatric phase I clinical trials (ADVL1513) the maximum tolerated dose (MTD) of entinostat for children was greater than for adults with no dose-limiting toxicity^17^. The preclinical study here demonstrates single agent activity or activity of entinostat in combination with current standard of care chemotherapeutics.

## Results

### The entinostat dosage approximating human AUC with a non-zero trough is 4 mg/kg po daily

**Figure 2:**
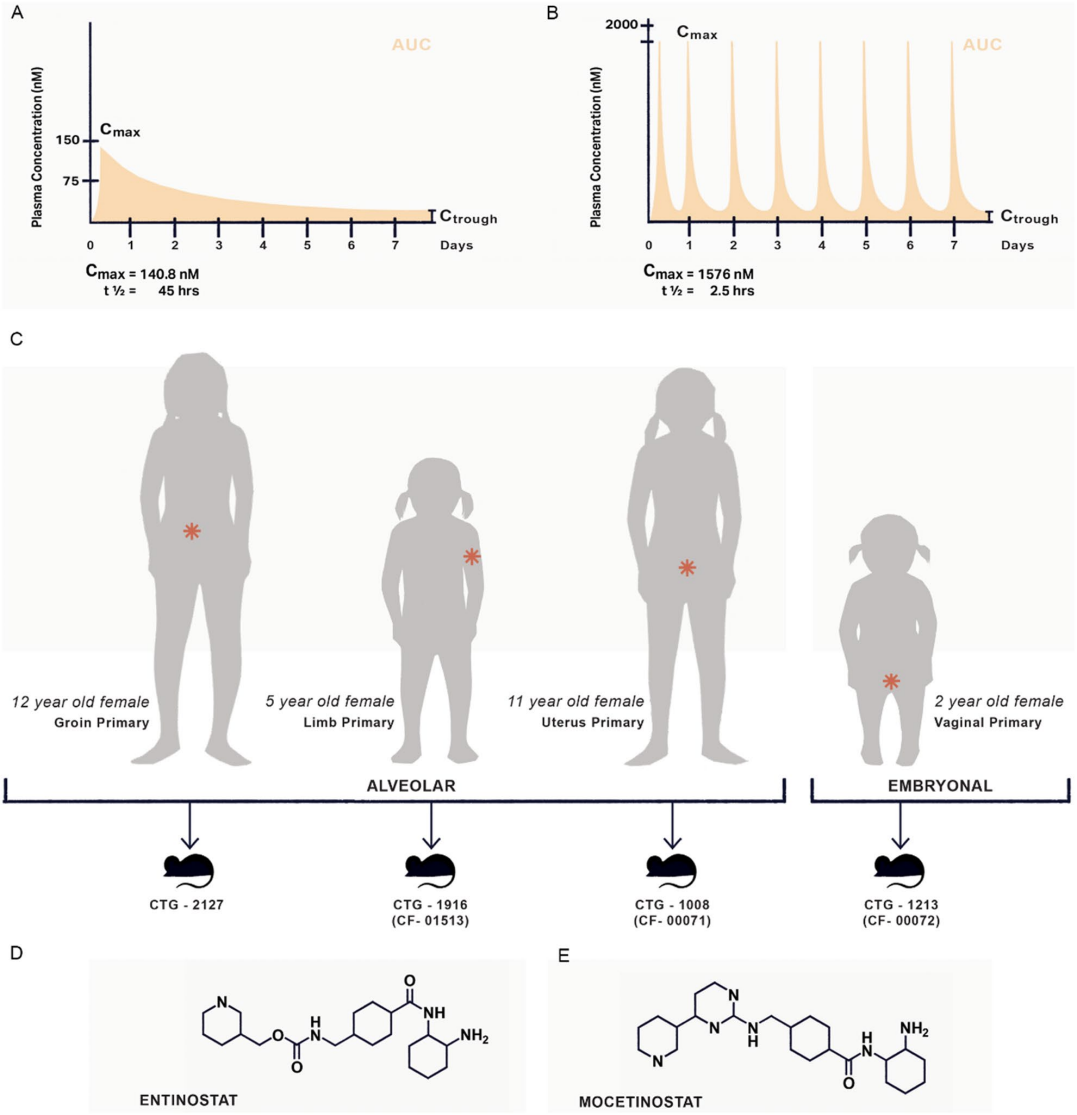
(**A**) Illustrative representation of entinostat pharmacokinetics in humans derived from clinical trial and preclinical data^17^. (**B**) Illustrative representation of entinostat pharmacokinetics in mice derived from clinical and preclinical data. (**C**) Patient derived xenograft mouse models of rhabdomyosarcoma (CTG-1008, CTG-1916, CTG-2127 and CTG1213). (**D**) Chemical structure of entinostat. (**E**) Chemical structure of mocetinostat. (illustration by Sun Young Park).

**Figure 3:**
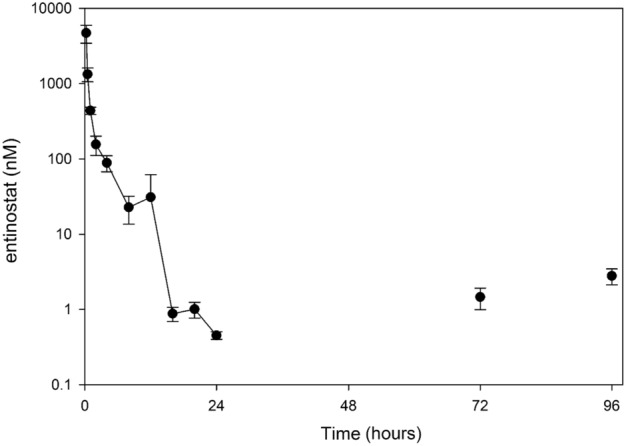
Concentration–time profiles of entinostat in mice (n = 3) treated with multiple doses of 4 mg/kg administered orally once daily. Plasma was obtained at timepoints over 24 h after a single dose as well as prior to the daily dose on Day 4 (72 h) and Day 5 (96 h). Data points and error bars represent mean and SD of 3 replicates, respectively.

The illustrative framework for drug dosing, PDX models and compounds tested is presented in Fig. 2A–E. In humans, pediatric patients given the recommended phase II dose (RP2D) of entinostat of 4 mg/m^2^ orally (po) administered once weekly have a C_max_ of 53 ng/mL (**140.8 nM**) with a t_1/2_ of 45 h, and a corresponding drug exposure (area under the curve, AUC) of 1162 ng*h/mL (3087 nM*h) (Fig. 2A)^17^. In mice, the plasma concentration–time profile following administration of a single dose or multiple doses of entinostat by oral gavage at 4 mg/kg is presented in Figs. 2B and 3. The C_max_ of 4710 ± 1260 nM occurred at 0.25 h. The AUC_0–12 h_ and AUC_0–24 h_ from single dose was 2470 nM*h and 2510 nM*h, respectively. The trough concentrations at 24, 72, and 96 h were 0.45 ± 0.05 nM, 1.46 ± 0.47 nM and 2.79 ± 0.66 nM, respectively. When correcting mouse exposure based on the value of unbound fraction of 0.38^18^, the C_max_ for unbound entinostat was **1576** ± 476 nM, and the AUC_0–12 h_ and AUC_0–24 h_ were 934 nM*h and 949 nM*h, respectively. **Trough concentrations at** 24, 72, and **96 h** were 0.17 ± 0.02 nM, 0.56 ± 0.18 nM and **1.06** ± 0.25 nM, respectively. In previous studies, a trough level > 1 nM was pivotal to maintaining PAX3::FOXO1 protein level suppression^3^. Therefore, prioritizing a murine AUC which is at or below the pediatric AUC and a ≥ 1 nM threshold trough level to model entinostat dosing between humans and mice, the oral gavage of 4 mg/kg was selected for combination studies in mice.

### The patient-derived xenograft models used in this study reflect stage IV and recurrent disease

Patient derived xenograft models were selected for implantation (Table 1). CTG-1008, CTG-1916, and CTG-2127 were selected as ARMS models, all featuring the *PAX3::FOXO1* fusion. No *PAX7::FOXO1* lines were available for implantation at the time of this study. CTG-1008 (alias CF-00071) was a recurrent stage IV tumor of the uterus biopsied from a 11-year-old female. CTG-1916 (alias CF-01513) was a recurrent stage IV tumor of the arm biopsied from a 5-year-old female. CTG-2127 was a stage IV tumor of the groin biopsied from a 12-year-old female. For comparison, CTG-1213 (alias CF-00072) was established from a biopsy of a 2-year-old female with stage IV ERMS of the vagina. CTG-1213 harbors an *NRAS* Q61K mutation. CTG-1213 served as a control for the effect of HDAC inhibition on a fusion negative tumor.

**Table 1:** Patient derived xenograft tumor demographics.

RMS model	Alias	Histology	Harvest site	Genetic features	Disease stage	Birth sex	Age (years)
CTG-1008	CF-0071	Rhabdomyosarcoma, alveolar	Uterus	PAX3::FOXO1	IV, recurrent	F	11
CTG-1916	CF-01513	Rhabdomyosarcoma, alveolar	Arm	PAX3::FOXO1	IV, recurrent	F	5
CTG-2127		Rhabdomyosarcoma, alveolar	Groin	PAX3::FOXO1	IV	F	12
CTG-1213	CF-00072	Rhabdomyosarcoma, embryonal, myxoid	Vagina	CREBBP::SLX4, CRAMP1::MAPK81P3	IV	F	2

### PDX models of ARMS respond to entinostat as a single agent or in combination with chemotherapies

**Figure 4:**
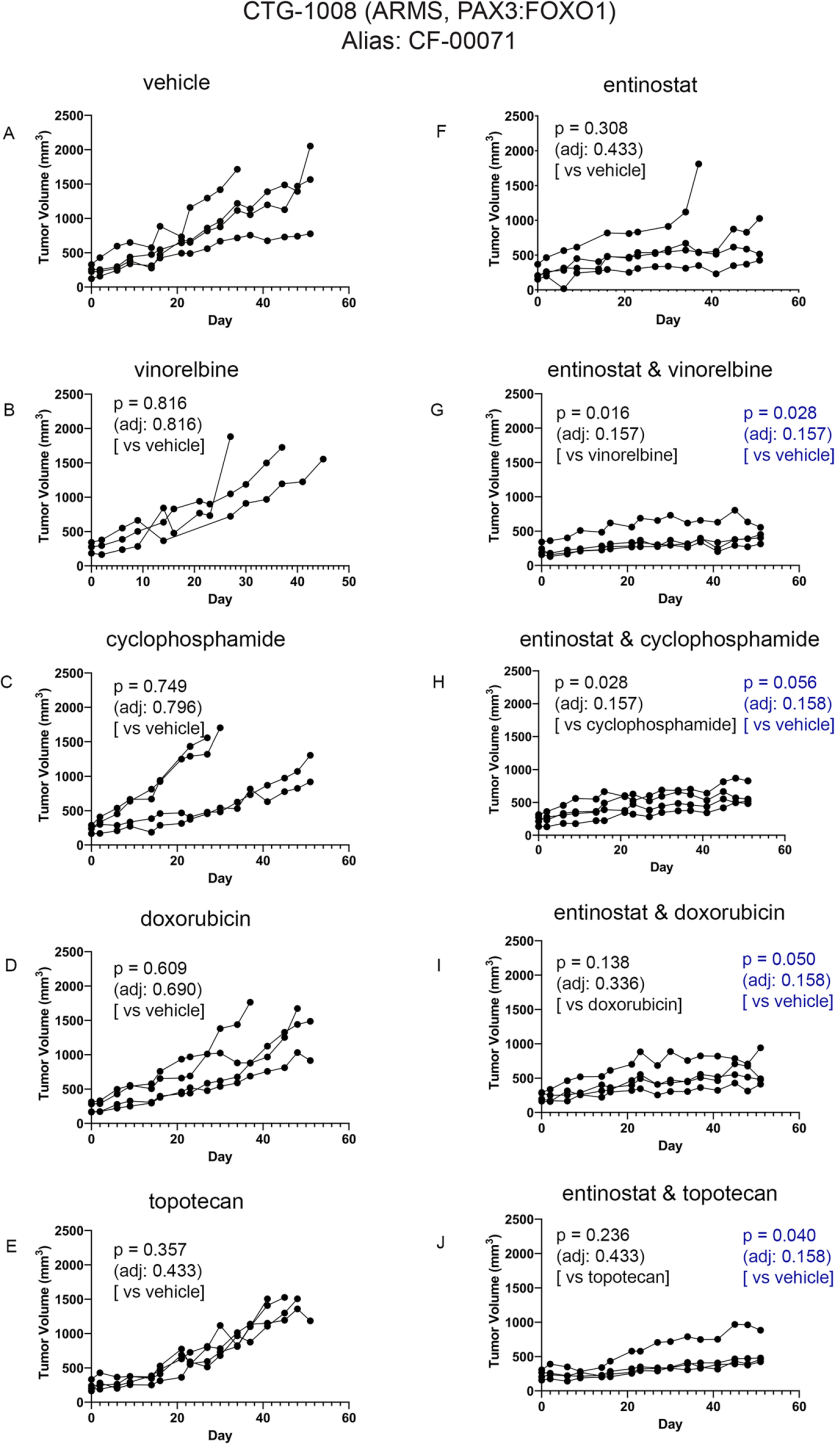
Impact of entinostat in combination with chemotherapies on tumor growth of ARMS PDX model CTG-1008. (**A**) Tumor growth of vehicle treated mice (n = 4). (**B**) Tumor growth of 4 mg/kg vinorelbine treated mice (n = 3). (**C**) Tumor growth of 50 mg/kg cyclophosphamide treated mice (n = 4). (**D**) Tumor growth of 2.5 mg/kg doxorubicin treated mice (n = 4). (**E**) Tumor growth of 0.15 mg/kg topotecan treated mice (n = 4). (**F**) Tumor growth of 4 mg/kg entinostat treated mice (n = 4). (**G**) Tumor growth of 4 mg/kg entinostat and 4 mg/kg vinorelbine treated mice (n = 4). (**H**) Tumor growth of 4 mg/kg entinostat and 50 mg/kg cyclophosphamide treated mice (n = 4). (**I**) Tumor growth of 4 mg/kg entinostat and 2.5 mg/kg doxorubicin treated mice (n = 4). (**J**) Tumor growth of 4 mg/kg entinostat and 0.15 mg/kg topotecan treated mice (n = 4). Each line represents one mouse’s tumor volume over time.

**Figure 5:**
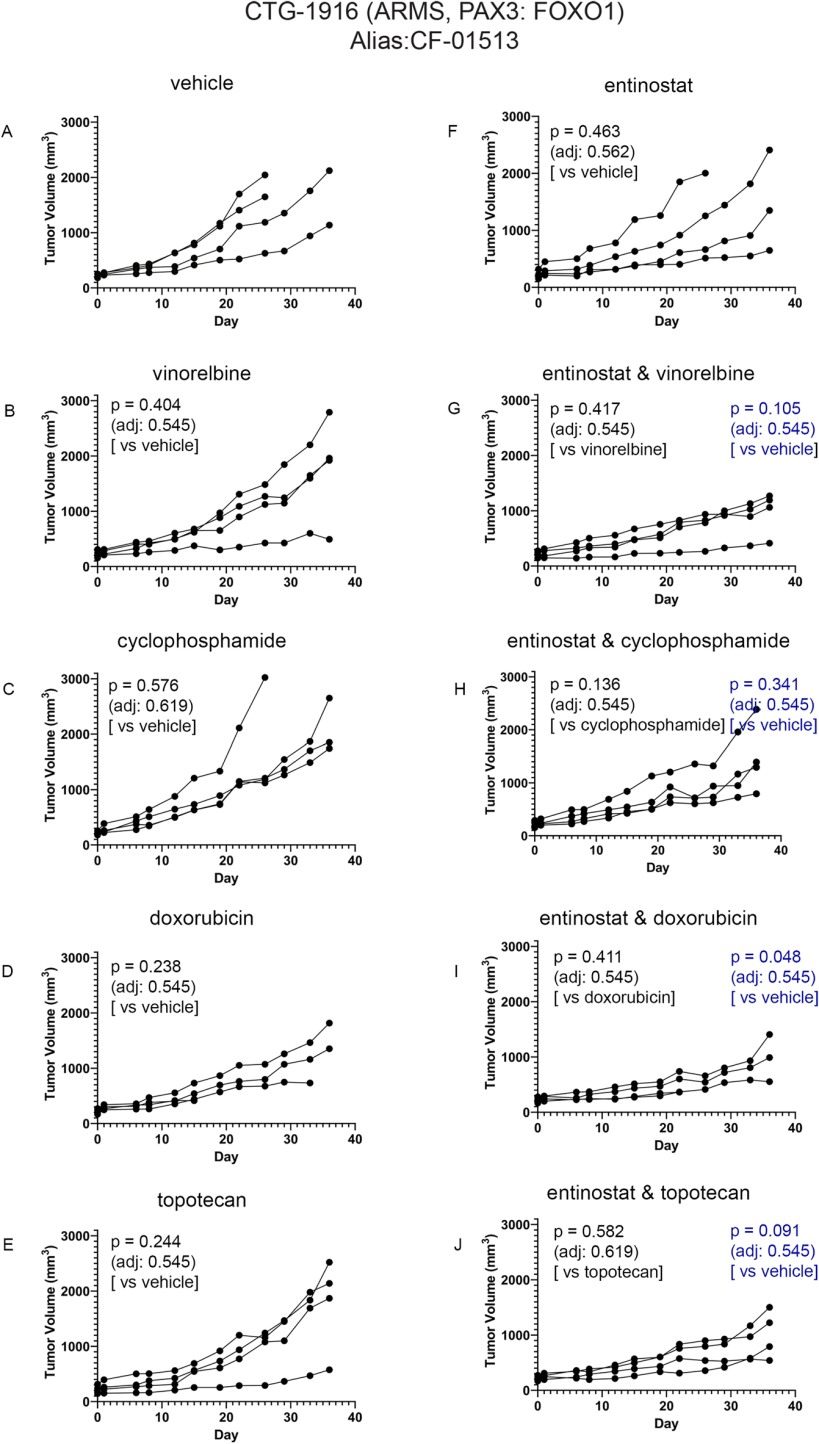
Impact of entinostat in combination with chemotherapies on tumor growth of ARMS PDX model CTG-1916. (**A**) Tumor growth of vehicle treated mice (n = 4). (**B**) Tumor growth of 4 mg/kg vinorelbine treated mice (n = 4). (**C**) Tumor growth of 50 mg/kg cyclophosphamide treated mice (n = 4). (**D**) Tumor growth of 3 mg/kg doxorubicin treated mice (n = 3). (**E**) Tumor growth of 0.15 mg/kg topotecan treated mice (n = 4). (**F**) Tumor growth of 4 mg/kg entinostat treated mice (n = 4). (**G**) Tumor growth of 4 mg/kg entinostat and 4 mg/kg vinorelbine treated mice (n = 4). (**H**) Tumor growth of 4 mg/kg entinostat and 50 mg/kg cyclophosphamide treated mice (n = 4). (**I**) Tumor growth of 4 mg/kg entinostat and 3 mg/kg doxorubicin treated mice (n = 3). (**J**) Tumor growth of 4 mg/kg entinostat and 0.15 mg/kg topotecan treated mice (n = 4). Each line represents one mouse’s tumor volume over time.

**Figure 6:**
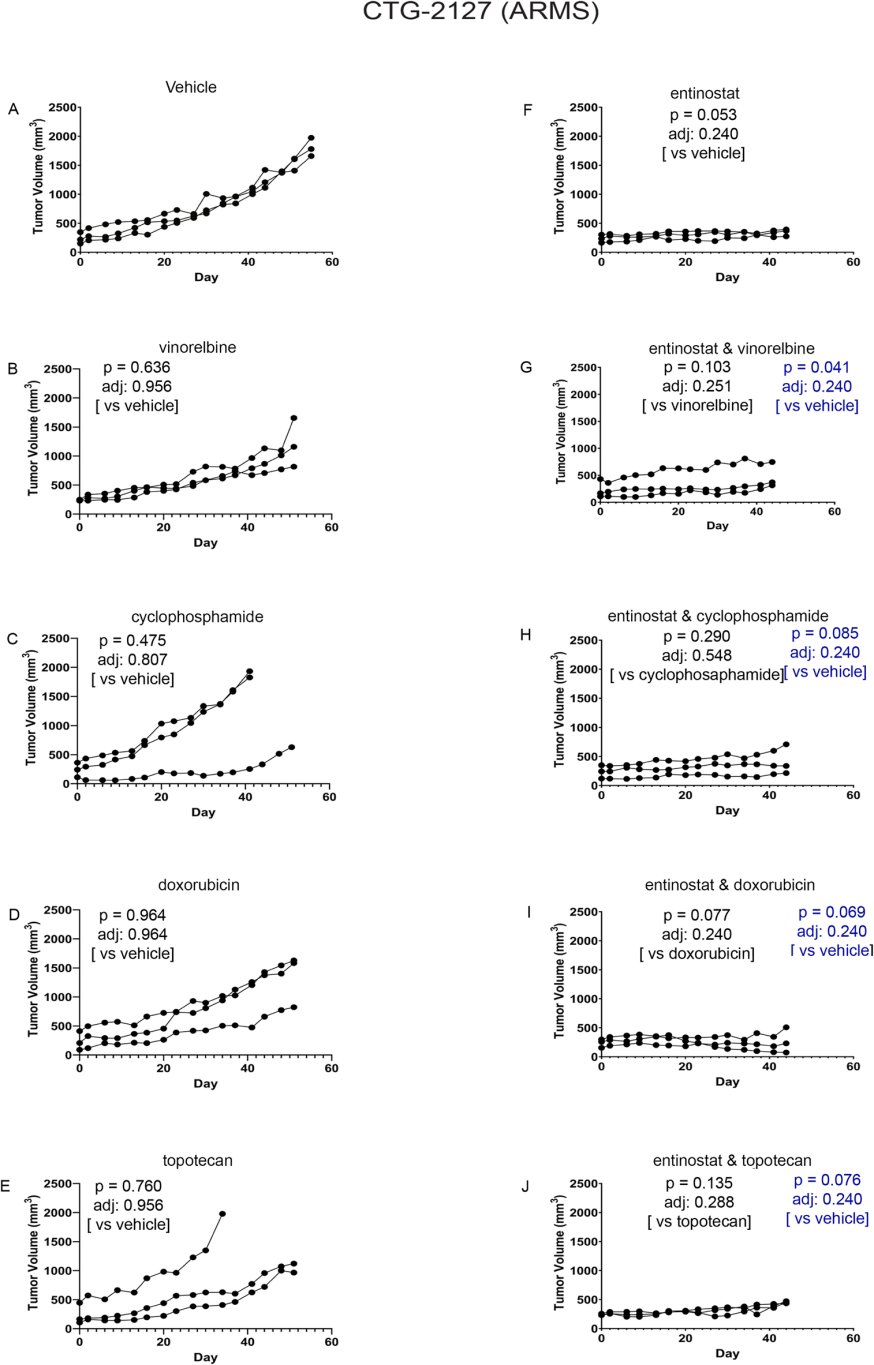
Impact of entinostat in combination with chemotherapies on tumor growth of ARMS PDX model CTG-2127. (**A**) Tumor growth of vehicle treated mice (n = 3). (B)Tumor growth of 4 mg/kg vinorelbine treated mice (n = 3). (**C**) Tumor growth of 50 mg/kg cyclophosphamide treated mice (n = 3). (**D**) Tumor growth of 2.5 mg/kg doxorubicin treated mice (n = 3). (**E**) Tumor growth of 0.15 mg/kg topotecan treated mice (n = 3). (**F**) Tumor growth of 4 mg/kg entinostat treated mice (n = 3). (**G**) Tumor growth of 4 mg/kg entinostat and 4 mg/kg vinorelbine treated mice (n = 3). (**H**) Tumor growth of 4 mg/kg entinostat and 50 mg/kg cyclophosphamide treated mice (n = 3). (**I**) Tumor growth of 4 mg/kg entinostat and 2.5 mg/kg doxorubicin treated mice (n = 3). (**J**) Tumor growth of 4 mg/kg entinostat and 0.15 mg/kg topotecan treated mice (n = 3). Each line represents one mouse’s tumor volume over time.

Single agent entinostat and entinostat-chemotherapy efficacy in tumor-bearing PDX models of ARMS are given Fig. 4 (CTG-1008), Fig. 5 (CTG-1916) and Fig. 6 (CTG-2127). The impact of treatment on body weight during the course of study is provided in Figures S1–S3. The entinostat dose and interval were guided by results from Fig. 3, which represents the plasma concentration–time profile following administration of a single dose of entinostat of 4 mg/kg in mice, whereas chemotherapy doses of vinorelbine, cyclophosphamide, doxorubicin and topotecan were selected intentionally to give an intermediate (non-curative) response so that the effect of entinostat could be observed and statistically tested. In general, all monotherapy and combination therapy regimens were well-tolerated. In Fig. 4, 5 and 6, one-way comparison p-values (single factor ANOVA analysis) are given for key comparisons of efficacy. Tables of complete statistical comparisons of efficacy and body weight monitoring for all PDX testing are given in Supplemental Tables 1–6.

For evaluation of entinostat monotherapy, models CTG-1008 (Fig. 4F) and CTG-1916 (Fig. 5F) had no single agent activity in line with previously-published studies showing that PAX3::FOXO1 is dispensable for tumor maintenance in the short term^4,19,20^. Unexpectedly, ARMS model CTG-2127 was exquisitely sensitive to entinostat monotherapy (Fig. 6F; p = 0.053 unadjusted).

For combination therapies, the exquisite sensitivity of CTG-2127 to monotherapy made evaluations of drug synergy not possible. However, for model CTG-1008, the combination of entinostat with vinorelbine and the combination of entinostat with cyclophosphamide reached statistical significance before adjusting for multiple comparisons (Fig. 4G, H; p = 0.016 and 0.028 unadjusted, respectively). Possible trends existed for the combination of entinostat with doxorubicin and the combination of entinostat with topotecan (Fig. 4I, J; p = 0.138 and 0.236 unadjusted, respectively). For model CTG-1916, a trend was present for the combination of entinostat with cyclophosphamide (Fig. 5H; p = 0.136 unadjusted).

### A single PDX model of ERMS responded modestly to entinostat as a single agent and in combination with chemotherapies

**Figure 7:**
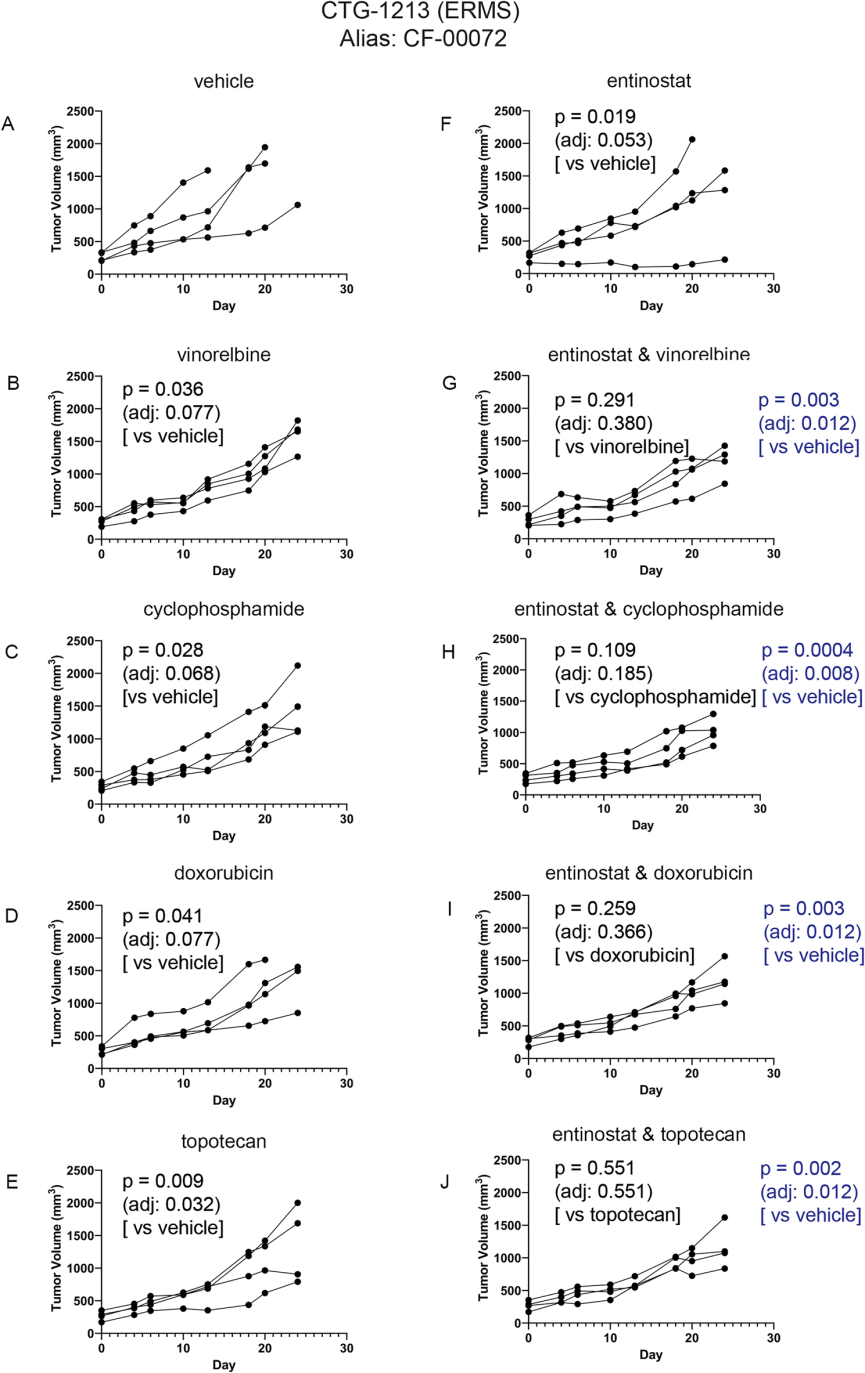
Impact of entinostat in combination with chemotherapies on tumor growth of ERMS PDX model CTG-1213. (**A**) Tumor growth of vehicle treated mice (n = 4). (**B**) Tumor growth of 4 mg/kg vinorelbine treated mice (n = 4). (**C**) Tumor growth of 50 mg/kg cyclophosphamide treated mice (n = 4). (**D**) Tumor growth of 2.5 mg/kg doxorubicin treated mice (n = 4). (**E**) Tumor growth of 0.15 mg/kg topotecan treated mice (n = 4). (**F**) Tumor growth of 4 mg/kg entinostat treated mice (n = 4). (**G**) Tumor growth of 4 mg/kg entinostat and 4 mg/kg vinorelbine treated mice (n = 4). (**H**) Tumor growth of 4 mg/kg entinostat and 50 mg/kg cyclophosphamide treated mice (n = 4). (**I**) Tumor growth of 4 mg/kg entinostat and 2.5 mg/kg doxorubicin treated mice (n = 4). (**J**) Tumor growth of 4 mg/kg entinostat and 0.15 mg/kg topotecan treated mice (n = 4). Each line represents one mouse’s tumor volume over time.

Single agent entinostat and entinostat-chemotherapy efficacy in a single tumor-bearing PDX model of ERMS is given Fig. 7 (CTG-1213). The impact of treatment on body weight during the course of study is provided in Fig. S4. As was used in ARMS PDX testing, the entinostat dose and interval were guided by results from Fig. 3, and chemotherapy doses were selected intentionally to give an intermediate response so that the effect of entinostat could be observed. As was used in ARMS PDX testing, monotherapy and combination therapy regimens were generally well-tolerated. In Fig. 7, one-way comparison p-values (single factor ANOVA analysis) are given for key comparisons of efficacy. Tables of complete statistical comparisons of efficacy and body weight monitoring for all PDX testing are given in Supplemental Tables 7, 8.

For evaluation of entinostat monotherapy, model CTG-1213 had a statistically significant response (Fig. 7F; p = 0.019 unadjusted). In previously published PDX studies, an entinostat monotherapy response was observed in 2 of 4 fusion negative RMS models^15^. For combination therapies, a trend was observed for the combination of entinostat with cyclophosphamide versus cyclophosphamide alone (Fig. 7H; p = 0.109 unadjusted) and the combination of entinostat with vinorelbine (Fig. 7G; p = 0.291 unadjusted).

### Entinostat and mocetinostat are mechanistically comparable with respect to reducing PAX3::FOXO1

Because the similar Class I/IV HDAC inhibitor, mocetinostat, has shown activity in combination with vinorelbine in an ongoing phase I clinical trial of patients with refractory or recurrent RMS^21^, we tested whether mocetinostat had similar mechanistic activity to decrease PAX3::FOXO1 protein. For the ARMS cell line Rh30 treated for 72 h, both entinostat and mocetinostat reduced PAX3::FOXO1 protein in a comparable, dose-dependent manner (Fig. S5).

## Discussion

Metastatic ARMS represents a significantly unmet clinical need. The challenge in treatment arises from chemoresistance, which is attributed to PAX3::FOXO1-mediated cell cycle checkpoint adaptation^3,4,22^. PAX3::FOXO1 can be targeted in the context of an HDAC3-SMARCA4-*miR-27a*-PAX3::FOXO1 circuit by HDAC3 inhibition^3^. The study presented here investigates the role of entinostat, a Class I/III HDAC inhibitor, as a single agent or in combination with 4 other chemotherapeutic drugs in 3 ARMS and 1 ERMS PDX models. Entinostat demonstrated efficacy when used as a single agent in one of the ARMS PDX models and in combination with other chemotherapeutics (vinorelbine, cyclophosphamide, doxorubicin and topotecan) for 2 of the 3 ARMS PDX models tested that were not already exquisitely sensitive to entinostat as a monotherapy. In the ERMS PDX model, entinostat exhibited a modest effect.

Pharmacokinetic modeling between humans and mice is often a challenge, which we addressed here with a clear rationale for the murine dose selected: we have compared the ADVL1513 pediatric phase I RP2D of 4 mg/m^2^ po weekly entinostat^17^ to the pharmacokinetics in the mouse at a dose of 4 mg/kg po daily, finding a shorter half-life in mice, a higher C_max_ in mice, a somewhat lower AUC in mice, yet a similar non-zero trough that supports the mechanism of entinostat to suppress PAX3::FOXO1 protein levels. The 4 mg/kg po daily dose is the same as we have published in previous preclinical studies for which 7 of 7 contemporary patient-derived xenografts (mostly autopsy-derived) showed additive or synergistic activity of entinostat in combination with the chemotherapy vincristine in vivo for ARMS^3^. For ERMS, single agent activity was observed for 2 of 4 contemporary patient-derived xenografts (mostly relapse-derived) in a previously reported study^15^. In contrast, another group’s study^23^ used a different dosing regimen and had notable design limitation: First, the pharmacodynamic studies were done with two alveolar rhabdomyosarcoma PDX models (Rh10 and Rh65) that lacked baseline PAX3::FOXO1 protein expression in 1 of 3 control animals for each model—drawing concerns for the authenticity of these models. In efficacy studies, only two ARMS PDX models (Rh10 and Rh41) were used, despite these models not being contemporary. Most importantly, for dosing (despite the known shorter murine half-life of entinostat) mice were given 3-day drug holidays per week and mouse dosing was only given 4 days per week at 2.5 mg/kg po BID. The lack of efficacy of entinostat plus vincristine in these mouse models was therefore not unexpected.

In our current study, entinostat monotherapy had no effect on tumor growth in 2 ARMS models, which is consistent with prior published observations that PAX3::FOXO1 is to a degree dispensable for tumor maintenance until PAX3::FOXO1 expression goes to absolute zero (*i.e.*, as seen comparing efficient RNA interference studies vs CRISPR)^4,19,20^ (depmap.org). However, in one ARMS model entinostat monotherapy had unexpected, near-complete efficacy to suppress tumor growth. For ERMS, one tested ERMS PDX model showed modest growth inhibition, which is in keeping with the ~ 50% response rate of fusion negative RMS that has been published^15^.

Despite PAX3::FOXO1 being dispensable in the short turn to tumor cell growth, PAX3::FOXO1 is nonetheless critical to mediate a G_2_-specific, Survivin/IAP-mediated process of chemotherapy resistance called cell cycle checkpoint adaptation^3–5,22^. Both genetic and pharmacological PAX3::FOXO1 inhibition induces significant chemotherapy sensitivity in vitro and in vivo^3,4,22^. As described in detail elsewhere, the underlying mechanism is that entinostat acts to inhibit a HDAC3-SMARCA4-*miR-27a*-PAX3::FOXO1 circuit (Fig. 1)^3,22^.

To test whether the synergies seen between entinostat, and vincristine extend to other rhabdomyosarcoma-specific, relapse-oriented chemotherapies, we tested entinostat with vinorelbine, cyclophosphamide, doxorubicin and topotecan in 3 ARMS PDX models and 1 ERMS PDX model. For ARMS, the exquisite sensitivity of one ARMS PDX model to entinostat abrogated evaluation of entinostat-mediated chemotherapy sensitization; however, in the 2 evaluable ARMS PDX models, a significant difference or trend was observed for the combination being greater than the efficacy of the single chemotherapy tested for each of the chemotherapies.

From a clinical trial concept perspective, correlative studies with ongoing rhabdomyosarcoma clinical trials are informative: the class I/IV HDAC inhibitor mocetinostat, has shown activity in combination with vinorelbine in an ongoing phase I clinical trial of patients with refractory or recurrent RMS^21^. For this study, enrollment on the dose escalation cohort has been completed and enrollment is ongoing for the dose expansion cohort. To date, 8 patients have been enrolled and 7 have had response evaluation. Of these 7 patients, 4 have had partial response (PR) and 2 have had stable disease (SD) for a disease control rate (DCR) of 86% with median duration of control of 8 months. To date, the only grade 3 or 4 treatment related adverse effects have been neutropenia, anemia and nausea. Neutropenia was transient and responsive to growth factors^21^. In our laboratory studies, mocetinostat and entinostat were comparable in the mechanistic effect of suppressing PAX3::FOXO1 levels. At present, mocetinostat is no longer accessible for clinical trials or commercial development, thereby restricting further examination in this population. Consequently, only entinostat was utilized in the murine PDX models.

On whole, these preclinical studies had the common challenge of matching human and murine pharmacokinetics, as well as the limitation of using only 3 ARMS PDX models and 1 ERMS PDX model. However, these studies are in keeping with extensive other PDX studies^3,15^, support a general observation that entinostat complements a range of chemotherapeutics, and show the mechanistic equivalency of entinostat and mocetinostat, another HDAC inhibitor with observed clinical efficacy in RMS. Taken together, these combined results support the ongoing development of entinostat for curative-intent clinical trials of entinostat plus chemotherapeutics for RMS.

## Materials and methods

### Pharmacokinetics

Mice were dosed with entinostat at 4 mg/kg or vehicle control via oral gavage once a day. Mice (n = 3/time point) were euthanized at 0.25, 0.5, 1, 2, 4, 8, 12, 16, 20 and 24 h after a single dose. Additional samples were obtained prior to the daily dose on Day 4 (72 h) and 5 (96 h). Entinostat was extracted from plasma (using sodium heparin) and analyzed by LC/MS/MS as previously described over the range of 0.3–266 nM with dilutions of 1:100 being accurate^24^. Pharmacokinetic parameters were calculated from mean concentration–time data using non-compartmental methods in Phoenix WinNonlin version 8.3 (Certara, Princeton, NJ). The maximum plasma concentration (C_max_) and time to C_max_ (T_max_) were the observed values. The AUC_0–12 h_ and AUC_0–24 h_ were calculated using the log-linear trapezoidal method.

### PDX models at Champions Oncology

All animal procedures were conducted at Champions Oncology in accordance with Institutional Animal Care and Use Committee (IACUC) protocols. The experimental protocols were reviewed and approved by the Institutional Animal Care and Use Committee (IACUC), Children’s Cancer Therapy Development Institute. The in vivo efficacy testing was performed using Champions’ TumorGraft models from three independent RMS biopsy specimens. The explants were received and immediately implanted into immunodeficient mice. Female nu/nu athymic nude mice (6–8 weeks old; Envigo, Indianapolis, Indiana) were utilized for the implantation. Anesthesia and analgesic were administered prior to surgery. A tumor fragment (~ 100 mm^3^) was implanted subcutaneously into the flank, according to standard operating procedures. Tumor dimensions were regularly measured with digital calipers, and tumor volume (TV) was calculated using the formula: TV = width^2^ × length × 0.52. Mice were randomized into study groups once the PDX tumor volumes reached approximately 150–300 mm^3^, at which point therapeutic dosing commenced.

The antitumor activity of entinostat in combination with cyclophosphamide, doxorubicin, topotecan, and vinorelbine was tested in a low-passage immune-compromised female mice Envigo nu/nu) at least 6 and 8 weeks of age, housed on irradiated virgin kraft sheet (Innorichment) and corncob bedding (Tekland), in individually-ventilated HEPA (high-efficiency particulate air) ventilated cages (Allentown, LLC) on a 14-h light/10-h dark cycle at 68° to 74 °F (20° to 23 °C) and 30–70% humidity. The animals were fed water ad libitum (reverse osmosis, 2 parts per million Cl2) and an irradiated test rodent diet (Tekland 2019) consisting of 19% protein, 9% fat, and 4% fiber. All compounds were formulated according to the manufacturer’s specifications (Table 2). Beginning day 0, tumor dimensions were measured twice weekly by a digital caliper, and data including individual and mean estimated tumor volumes (mean TV ± SEM) were recorded for each group; tumor volume was calculated using the following formula (1): TV = width^2^ × length × 0.52.

**Table 2:** Reagents and therapeutics used in this study.

Component	Catalog number	Vendor	Location
Hydrochloride (HCl)	320331	Sigma-Aldrich	Milwaukee, WI
Tween-80	P1754	Sigma-Aldrich	Milwaukee, WI
Sterile water	SH30221.LS	Cytiva	Logan, UT
Entinostat (MS-275)	S1053	Selleckchem	Houston, TX
Mocetinostat (MGCD0103)	sc-364539	Santa Cruz Biotechnology, Inc	Dallas, TX
Cyclophosphamide	0781-3244-94	Manufacturer: Sandoz Inc, Pharmacy: Blue door pharma	Pharmacy: Rockville, MD
Doxorubicin	0069-3032-20	Manufacturer: Pfizer, Pharmacy: Blue door pharma	Pharmacy: Rockville, MD
Vinorelbine	25021-204-01	Manufacturer: Sagent Pharmaceuticals, Pharmacy: Blue Door Pharma	Pharmacy: Rockville, MD
Topotecan	0409-0302-01	Manufacturer: Hospira Inc., Pharmacy: Blue Door Pharma	Pharmacy: Rockville, MD

Drug and vehicle sources and doses by model are given in Tables 2 and 3, respectively. In some cases, chemotherapy doses were reduced due to transient body weight loss. Dose volume was 10 mL/kg for all agents. The study endpoint was mean tumor volume of the control group reaching 1500 mm^3^. When this occurred before Day 28, treatment groups and individual mice were dosed and measured up to Day 28. When the mean tumor volume of the control group (uncensored) did not reach 1500 mm^3^ by Day 28, then the endpoint for all animals was the day when the mean tumor volume of the control group (uncensored) reached 1500 mm^3^ up to a maximum of Day 60. Individual mice reporting a tumor volume > 120% of the day 0 measurement a were considered to have progressive disease (PD).

**Table 3:** Drug doses by model.

Component/RMS model	CTG-1008 (Alias: CF-00071)	CTG-1916 (Alias: CF-01513)	CTG-2127	CTG-1213 (Alias: CF-00072)
Vehicle (0.05 HCl & 0.1% Tween 80 in sterile water)	PO/QD × 28	PO/QD × 28	PO/QD × 28	PO/QD × 28
Entinostat	4 mg/kg, PO/QD × 28	4 mg/kg, PO/QD × 28	4 mg/kg, PO/QD × 28	4 mg/kg, PO/QD × 28
Vinorelbine	4 mg/kg, IP/Q7D × 2	4 mg/kg, IP/Q7D × 2	4 mg/kg, IP/Q7D × 2	4 mg/kg, IP/Q7D × 2
Cyclophosphamide	50 mg/kg, IP/QD × 1	50 mg/kg, IP/QD × 1	50 mg/kg, IP/QD × 1	50 mg/kg, IP/QD × 1
Doxorubicin	2.5 mg/kg, IP/QD × 2	3 mg/kg, IP/QD × 2	2.5 mg/kg, IP/QD × 2	2.5 mg/kg, IP/QD × 2
Topotecan	0.15 mg/kg, IP/QD × 5	0.15 mg/kg, IP/QD × 5	0.15 mg/kg, IP/QD × 5	0.15 mg/kg, IP/QD × 5

### Statistics

The significance of variation in the mean of the tumor volumes comparing two groups with treatment was assessed with a repeated measures mixed effects linear model with an autoregressive order 1 correlation matrix. All statistical testing was two-sided. A Benjamini–Hochberg correction was applied to control the False Discovery Rate at 5%. SAS (SAS Institute, Cary, NC) and R (R Foundation for Statistical Computing, Vienna, Austria) were used throughout.

### Mice weights

Animals were weighed twice weekly. A final weight was taken on the day the study reached end point or when the animal was found moribund. Animals exhibiting > 10% weight loss when compared to Day 0 were provided DietGel® ad libitum. Any animal exhibiting > 20% net weight loss for a period lasting 7 days or when mice display > 30% net weight loss when compared to Day 0 were considered moribund and euthanized. Due to consistent weight loss observed in animals in Groups 4, 5, 8 and 9 (doxorubicin and vinorelbine treated groups) DietGel was administered on the same day as dosing on Day 0 and continued for the remainder of the study.

### PAX3::FOXO1 immunodetection for Rh30 treated with mocetinostat and entinostat

Pax3:FOXO1 positive cell line Rh30 was cultured in T25 cell culture flasks (cat. 163371, Thermo Fisher Scientific, Waltham, MA). The cells were incubated overnight in RPMI 1640 Medium (cat. 11875093, Thermo Fisher Scientific) supplemented with 10% fetal bovine serum (FBS) (cat. 26140079, Thermo Fisher Scientific) and 1% penicillin (100 U/mL)/streptomycin (100 μg/mL) (cat. 15140-122, Thermo Fischer Scientific) in a 37 °C incubator, supplemented with 5% CO_2_.

Once the cells reached 60–70% confluency, they were dosed with entinostat (cat. S1053, Selleck Chem, Houston, TX) and mocetinostat (cat. sc-364539, Santa Cruz Biotechnology, Dallas, TX) at the concentrations of 0, 0.2, 0.4 and 0.8 μM. The cells were incubated for 72 h and then harvested.

Cell lysates were collected after 72 h using 1:100 dilution radioimmunoprecipitation assay (RIPA) lysis buffer (cat. 89900, Thermo Fischer Scientific) with halt protease and phosphatase inhibitor cocktail (cat. 78440, Thermo Fisher Scientific) and analyzed for PAX3::FOXO1 expression using anti-PAX3/PAX7 antibody (1:50, cat. MAB2457, R&D Systems, Minneapolis, MN 55413) and matched for protein expression using a GAPDH antibody (1:15000, cat. 2118, Cell Signaling Technology, Danvers, MA 01923). The blot was visualized using a JESS Automated Western Blot System (ProteinSimple, San Jose, CA). This experiment was conducted thrice.

### Compliance with ethical standards

All animal studies were conducted with de-identified human cells/tissue and all experimental procedures were performed according to the guidelines of the Animal Research: Reporting of In Vivo Experiments (ARRIVE) and Institutional Animal Care and Use Committee (IACUC) of Champions Oncology.

### Supplementary Information

Supplementary Information.

## Data Availability

All data generated or analyzed during this study are included in the paper or supplementary material. No sequencing data was generated by this study.
