# Supplementary material for: Entinostat as a combinatorial therapeutic for rhabdomyosarcoma
Source: Sci Rep. 2024 Aug 15;14:18936. doi: 10.1038/s41598-024-66545-5 (PMC11327338; doi:10.1038/s41598-024-66545-5)
Supplement: Supplementary file 1 — Supplementary Information. [file 41598_2024_66545_MOESM1_ESM.docx]

**Supplemental Materials**

**Supplemental Figure Legends**

**Supplementary Figure S1 (relates to Figure 3).** Impact of treatment on body weight of PDX model CTG-1008. (A) Body weights of vehicle treated mice (n=4). (B) Body weights of 4 mg/kg vinorelbine treated mice (n=3). (C) Body weights of 50 mg/kg cyclophosphamide treated mice (n= 4). (D) Body weights of 2.5 mg/kg doxorubicin treated mice (n=4). (E) Body weights of 0.15 mg/kg topotecan treated mice (n=4). (F) Body weights of 4 mg/kg entinostat treated mice (n=4). (G) Body weights of 4 mg/kg entinostat and 4 mg/kg vinorelbine treated mice (n=4). (H) Body weights of 4 mg/kg entinostat and 50 mg/kg cyclophosphamide treated mice (n=4). (I) Body weights of 4 mg/kg entinostat and 2.5 mg/kg doxorubicin treated mice (n=4). (J) Body weights of 4 mg/kg entinostat and 0.15 mg/kg topotecan treated mice (n=4). Each line represents one mouse’s body weight over the course of the study.

**Supplementary Figure S2 (relates to Figure 4)**. Impact of treatment on body weight of PDX model CTG-1916. (A) Body weights of vehicle treated mice (n=4). (B) Body weights of 4 mg/kg vinorelbine treated mice (n=4). (C) Body weights of 50 mg/kg cyclophosphamide treated mice (n= 4). (D) Body weights of 3 mg/kg doxorubicin treated mice (n=3). (E) Body weights of 0.15 mg/kg topotecan treated mice (n=4). (F) Body weights of 4 mg/kg entinostat treated mice (n=4). (G) Body weights of 4 mg/kg entinostat and 4 mg/kg vinorelbine treated mice (n=4). (H) Body weights of 4 mg/kg entinostat and 50 mg/kg cyclophosphamide treated mice (n=4). (I) Body weights of 4 mg/kg entinostat and 3 mg/kg doxorubicin treated mice (n=3). (J) Body weights of 4 mg/kg entinostat and 0.15 mg/kg topotecan treated mice (n=4). Each line represents one mouse’s body weight over the course of the study.

**Supplementary Figure S3 (relates to Figure 5).** Impact of treatment on body weight of PDX model CTG-2127. (A) Body weights of vehicle treated mice (n=3). (B) Body weights of 4 mg/kg vinorelbine treated mice (n=3). (C) Body weights of 50 mg/kg cyclophosphamide treated mice (n= 3). (D) Body weights of 2.5 mg/kg doxorubicin treated mice (n=3). (E) Body weights of 0.15 mg/kg topotecan treated mice (n=3). (F) Body weights of 4 mg/kg entinostat treated mice (n=3). (G) Body weights of 4 mg/kg entinostat and 4 mg/kg vinorelbine treated mice (n=3). (H) Body weights of 4 mg/kg entinostat and 50 mg/kg cyclophosphamide treated mice (n=3). (I) Body weights of 4 mg/kg entinostat and 2.5 mg/kg doxorubicin treated mice (n=3). (J) Body weights of 4 mg/kg entinostat and 0.15 mg/kg topotecan treated mice (n=3). Each line represents one mouse’s body weight over the course of the study.

**Supplementary Figure S4 (relates to Figure 6).** Impact of treatment on body weight of PDX model CTG-1213. (A) Body weights of vehicle treated mice (n=4). (B) Body weights of 4 mg/kg vinorelbine treated mice (n=4). (C) Body weights of 50 mg/kg cyclophosphamide treated mice (n=4). (D) Body weights of 2.5 mg/kg doxorubicin treated mice (n=4). (E) Body weights of 0.15 mg/kg topotecan treated mice (n=4). (F) Body weights of 4 mg/kg entinostat treated mice (n=4). (G) Body weights of 4 mg/kg entinostat and 4 mg/kg vinorelbine treated mice (n=4). (H) Body weights of 4 mg/kg entinostat and 50 mg/kg cyclophosphamide treated mice (n=4). (I) Body weights of 4 mg/kg entinostat and 2.5 mg/kg doxorubicin treated mice (n=4). (J) Body weights of 4 mg/kg entinostat and 0.15 mg/kg topotecan treated mice (n=4). Each line represents one mouse’s body weight over the course of the study.

**Supplementary Figure S5.** Comparative study of entinostat and mocetinostat in Rh30 (ARMS) cell line measuring inhibition of PAX3::FOXO1 expression. (A-B) Chemiluminescence showing PAX3::FOXO1 expression with varying entinostat concentrations (A) and mocetinostat concentrations (B). (C) Virtual western blot derived from capillary immunoblot for PAX3::FOXO1 expression with increasing entinostat and mocetinostat concentrations. (D-E) Quantified expression of PAX3::FOXO1 in Rh30 (ARMS) cell line with increasing concentrations of entinostat (D) and mocetinostat (E).

**Figure S1**


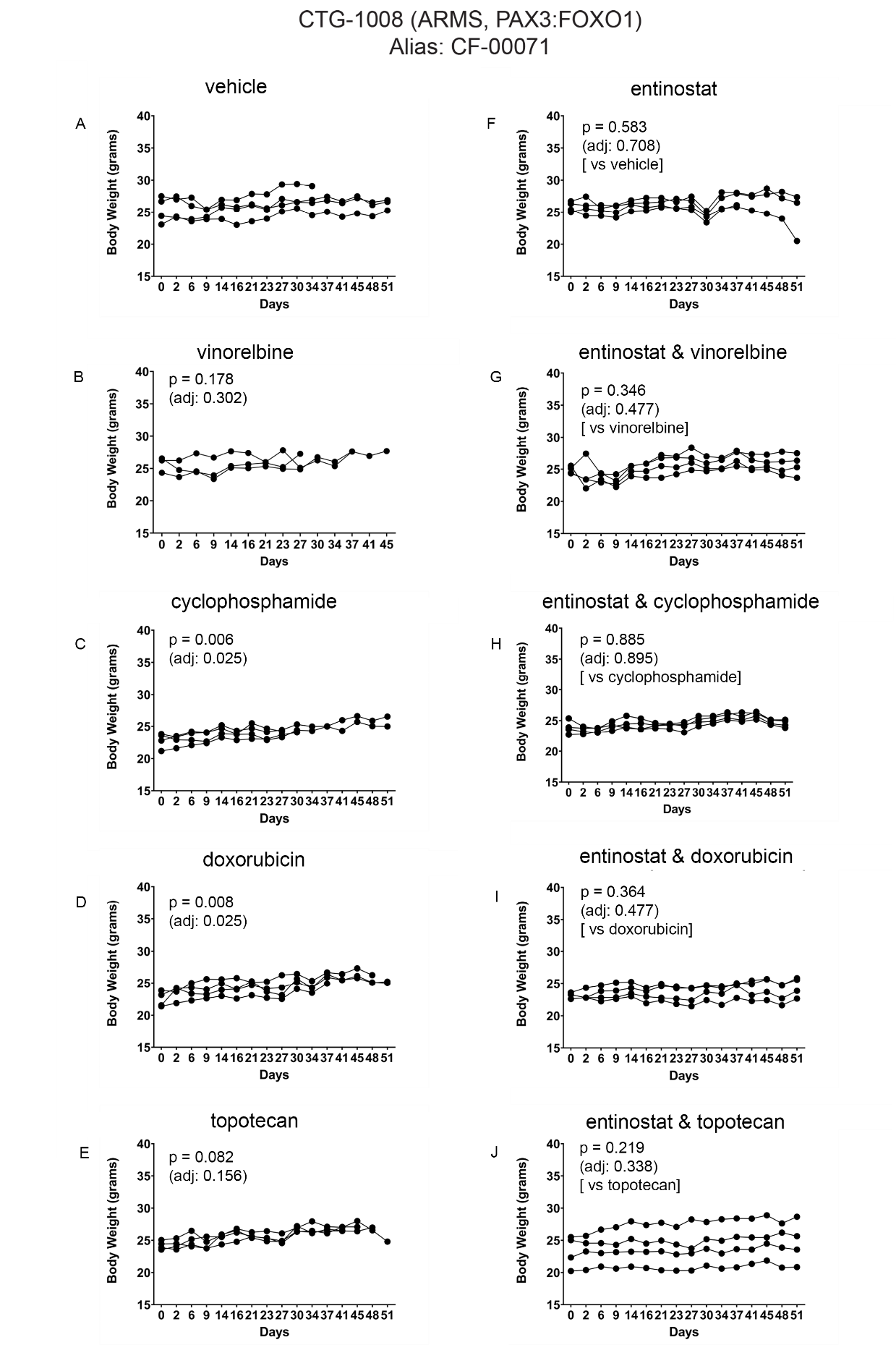


**Figure S2**


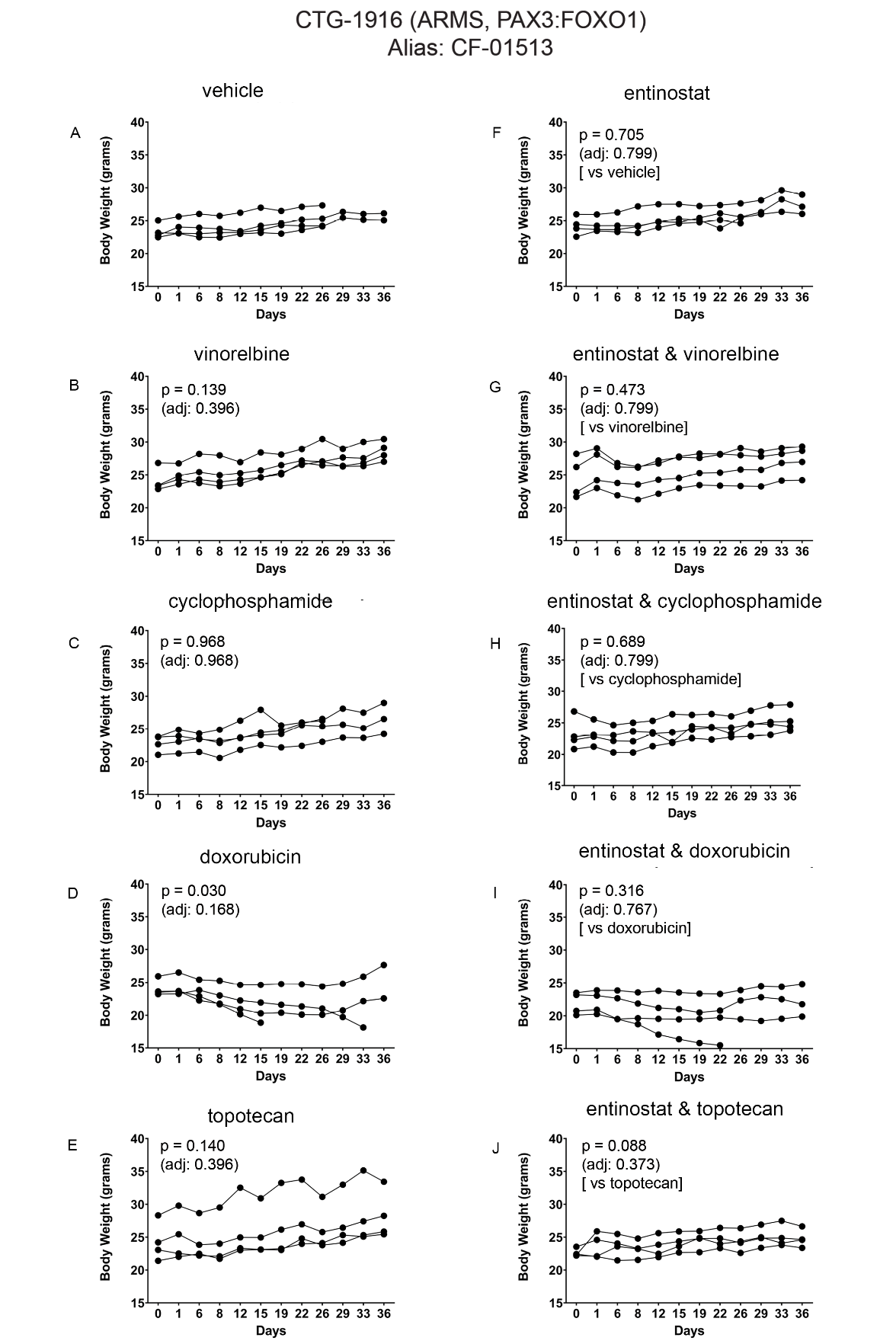


**Figure S3**


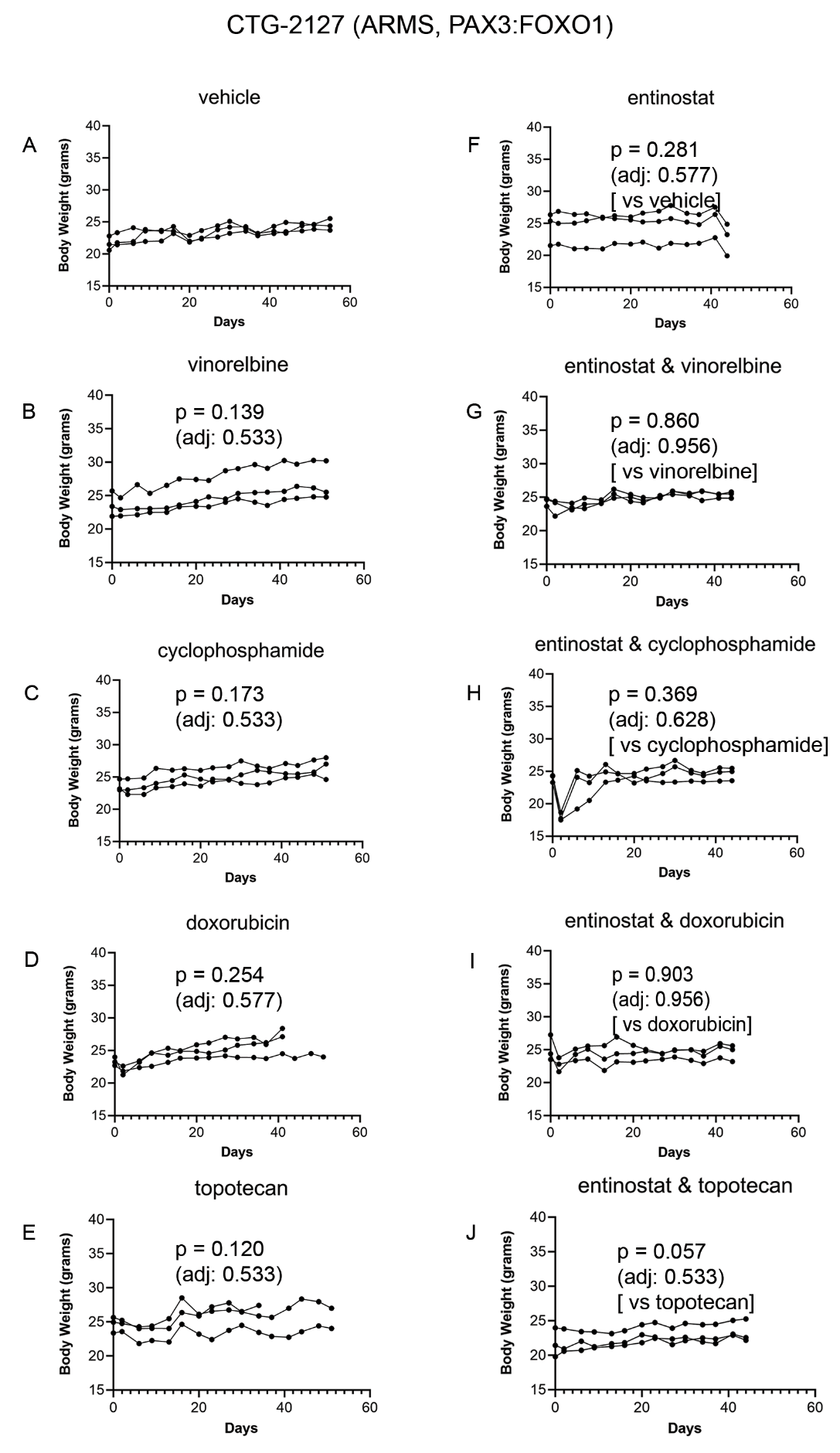


**Figure S4**


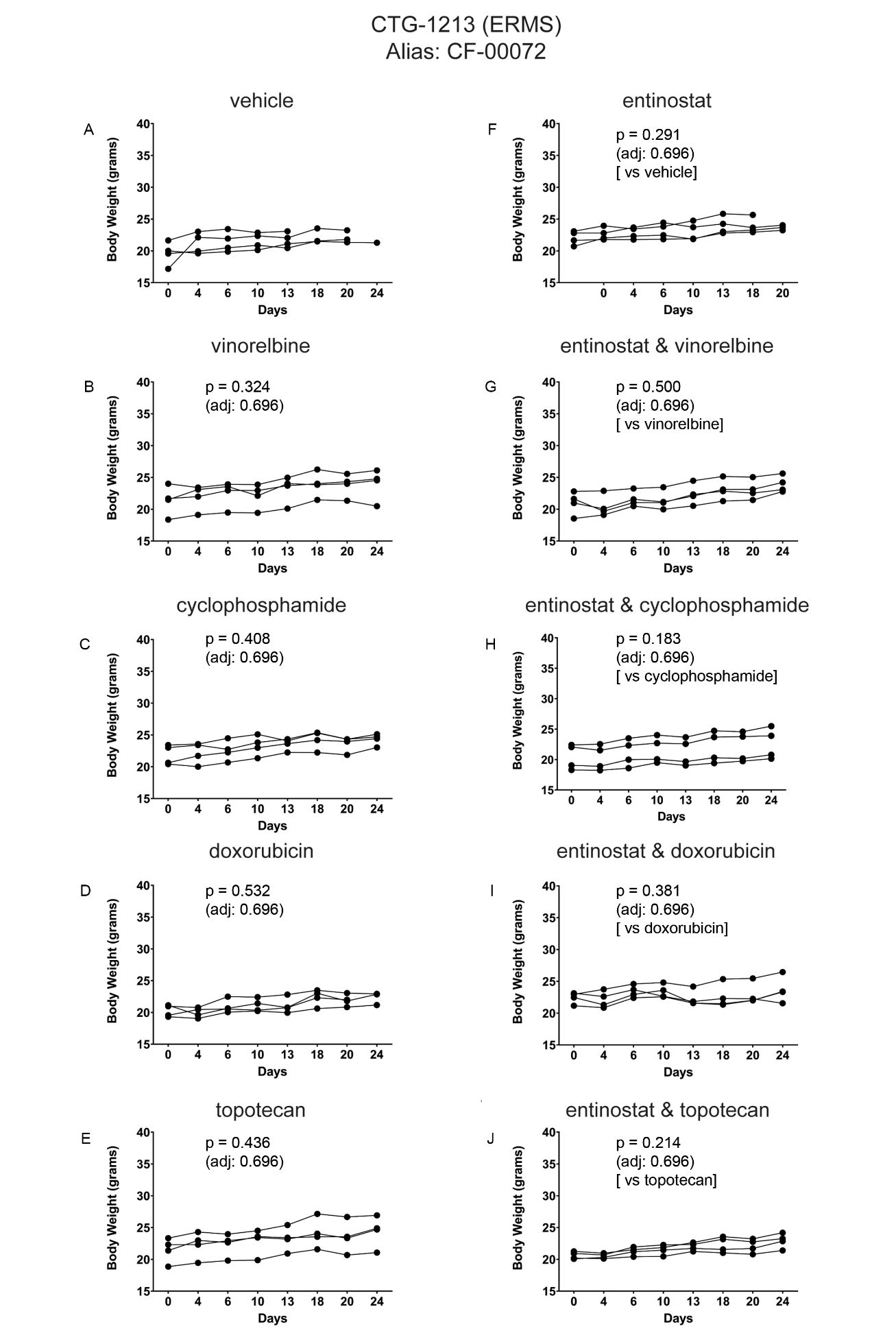


**Figure S5**


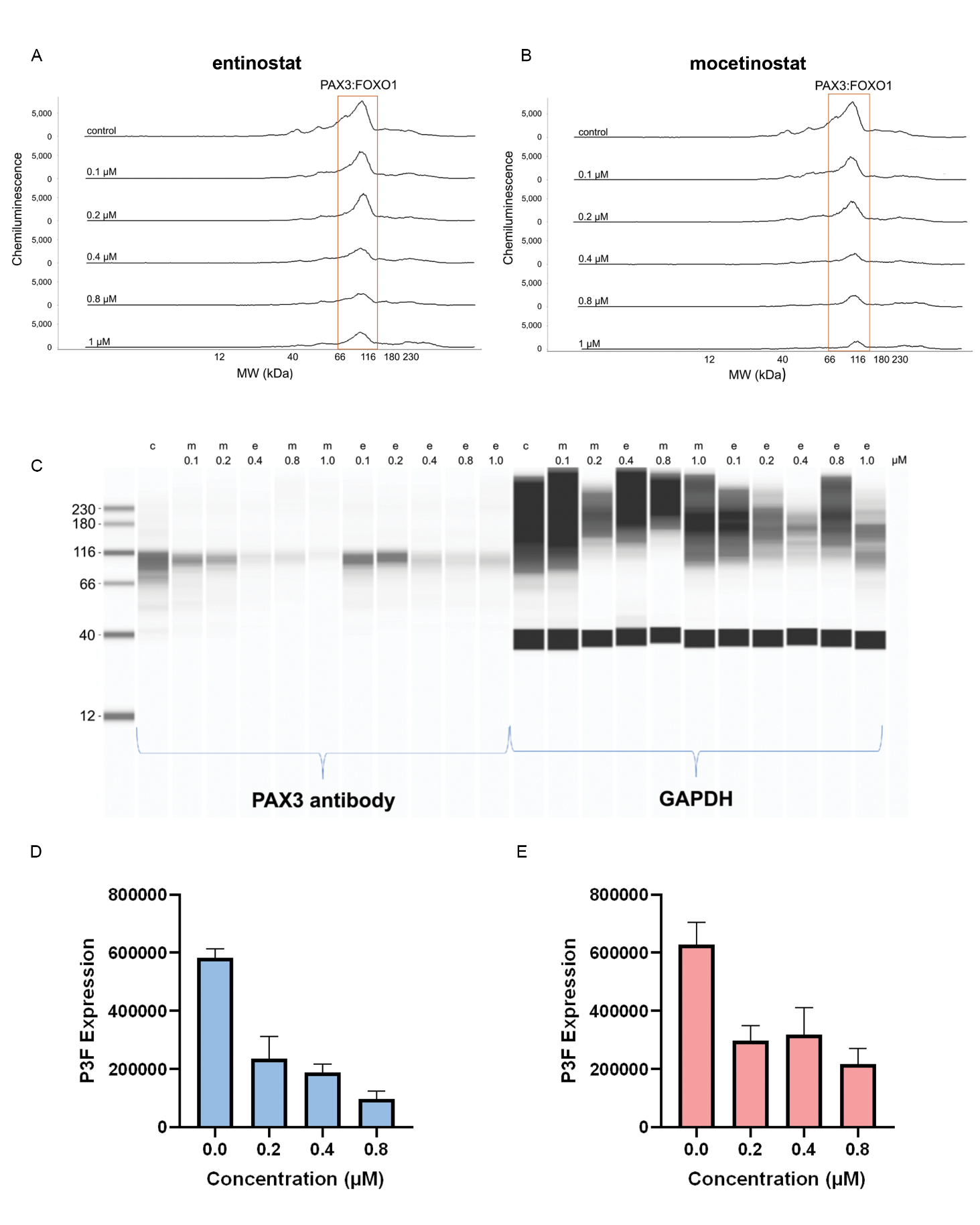


**Supplemental Tables**

**Supplemental Table 1. Statistics for Tumor volume of CTG-1008**

| group | diff | se | lower | upper | t | df | pv | adjusted.p |
| --- | --- | --- | --- | --- | --- | --- | --- | --- |
| Control vs Entinostat 4 mg/kg | 226.25 | 218.2333 | -219.4418907 | 671.9419 | 1.0367345 | 30 | 0.3081443 | 0.4332393 |
| Control vs Cyclophosphamide 50 mg/kg | -70.50 | 218.2333 | -516.1918907 | 375.1919 | -0.3230488 | 30 | 0.7488993 | 0.7957055 |
| Control vs Doxorubicin 2.5 mg/kg | 112.75 | 218.2333 | -332.9418907 | 558.4419 | 0.5166489 | 30 | 0.6091892 | 0.6904145 |
| Control vs Vinorelbine 4 mg/kg | -51.25 | 218.2333 | -496.9418907 | 394.4419 | -0.2348404 | 30 | 0.8159282 | 0.8159282 |
| Control vs Topotecan 0.15 mg/kg | 204.25 | 218.2333 | -241.4418907 | 649.9419 | 0.9359249 | 30 | 0.3567853 | 0.4332393 |
| Control vs Entinostat 4 mg/kg + Cyclophosphamide | 434.25 | 218.2333 | -11.4418907 | 879.9419 | 1.9898428 | 30 | 0.0557907 | 0.1580737 |
| 50 mg/kg  Control vs Entinostat 4 mg/kg + Doxorubicin 2.5 mg/kg | 445.00 | 218.2333 | -0.6918907 | 890.6919 | 2.0391020 | 30 | 0.0503345 | 0.1580737 |
| Control vs Entinostat 4 mg/kg + Vinorelbine 4 mg/kg | 505.00 | 218.2333 | 59.3081093 | 950.6919 | 2.3140371 | 30 | 0.0276978 | 0.1573577 |
| Control vs Entinostat 4 mg/kg + Topotecan 0.15 mg/kg | 468.00 | 218.2333 | 22.3081093 | 913.6919 | 2.1444938 | 30 | 0.0402140 | 0.1580737 |
| Entinostat 4 mg/kg vs Entinostat 4mg/kg + Cyclophosphamide 50 mg/kg | 208.00 | 218.2333 | -237.6918907 | 653.6919 | 0.9531084 | 30 | 0.3481520 | 0.4332393 |
| Entinostat 4 mg/kg vs Entinostat 4 mg/kg + Doxorubicin 2.5 mg/kg | 218.75 | 218.2333 | -226.9418907 | 664.4419 | 1.0023676 | 30 | 0.3241830 | 0.4332393 |
| Entinostat 4 mg/kg vs Entinostat 4 mg/kg + Vinorelbine 4 mg/kg | 278.75 | 218.2333 | -166.9418907 | 724.4419 | 1.2773027 | 30 | 0.2112926 | 0.4332393 |
| Entinostat 4 mg/kg vs Entinostat 4 mg/kg + Topotecan 0.15 mg/kg | 241.75 | 218.2333 | -203.9418907 | 687.4419 | 1.1077594 | 30 | 0.2767686 | 0.4332393 |
| Cyclophosphamide 50 mg/kg vs Entinostat 4 mg/kg + Cyclophosphamide 50 mg/kg | 504.75 | 218.2333 | 59.0581093 | 950.4419 | 2.3128916 | 30 | 0.0277690 | 0.1573577 |
| Doxorubicin 2.5 mg/kg vs Entinostat 4 mg/kg + Doxorubicin 2.5 mg/kg | 332.25 | 218.2333 | -113.4418907 | 777.9419 | 1.5224532 | 30 | 0.1383679 | 0.3360363 |
| Vinorelbine 4 mg/kg vs Entinostat 4 mg/kg + Vinorelbine 4 mg/kg | 556.25 | 218.2333 | 110.5581093 | 1001.9419 | 2.5488775 | 30 | 0.0161615 | 0.1573577 |
| Topotecan 0.15 mg/kg vs Entinostat 4 mg/kg + Topotecan 0.15 mg/kg | 263.75 | 218.2333 | -181.9418907 | 709.4419 | 1.2085689 | 30 | 0.2362687 | 0.4332393 |

3

**Supplemental Table 2. Statistics for Body weights of CTG-1008**

| group | diff | se | lower | upper | t | df | pv | adjusted.p |
| --- | --- | --- | --- | --- | --- | --- | --- | --- |
| Control vs Entinostat 4 mg/kg | 0.5625 | 1.012544 | -1.5053907 | 2.630391 | 0.5555314 | 30 | 0.5826507 | 0.7075044 |
| Control vs Cyclophosphamide 50 mg/kg | 2.9850 | 1.012544 | 0.9171093 | 5.052891 | 2.9480200 | 30 | 0.0061401 | 0.0249402 |
| Control vs Doxorubicin 2.5 mg/kg | 2.8650 | 1.012544 | 0.7971093 | 4.932891 | 2.8295067 | 30 | 0.0082355 | 0.0249402 |
| Control vs Vinorelbine 4 mg/kg | 1.3975 | 1.012544 | -0.6703907 | 3.465391 | 1.3801869 | 30 | 0.1777282 | 0.3021380 |
| Control vs Topotecan 0.15 mg/kg | 1.8200 | 1.012544 | -0.2478907 | 3.887891 | 1.7974528 | 30 | 0.0823371 | 0.1555257 |
| Control vs Entinostat 4 mg/kg + Cyclophosphamide 50 mg/kg | 2.8375 | 1.012544 | 0.7696093 | 4.905391 | 2.8023474 | 30 | 0.0088024 | 0.0249402 |
| Control vs Entinostat 4 mg/kg + Doxorubicin 2.5 mg/kg | 3.7975 | 1.012544 | 1.7296093 | 5.865391 | 3.7504543 | 30 | 0.0007544 | 0.0128248 |
| Control vs Entinostat 4 mg/kg + Vinorelbine 4 mg/kg | 0.4275 | 1.012544 | -1.6403907 | 2.495391 | 0.4222039 | 30 | 0.6758868 | 0.7660050 |
| Control vs Entinostat 4 mg/kg + Topotecan 0.15 mg/kg | 3.0925 | 1.012544 | 1.0246093 | 5.160391 | 3.0541883 | 30 | 0.0047009 | 0.0249402 |
| Entinostat 4 mg/kg vs Entinostat 4mg/kg + Cyclophosphamide 50 mg/kg | 2.2750 | 1.012544 | 0.2071093 | 4.342891 | 2.2468160 | 30 | 0.0321648 | 0.0683501 |
| Entinostat 4 mg/kg vs Entinostat 4 mg/kg + Doxorubicin 2.5 mg/kg | 3.2350 | 1.012544 | 1.1671093 | 5.302891 | 3.1949229 | 30 | 0.0032812 | 0.0249402 |
| Entinostat 4 mg/kg vs Entinostat 4 mg/kg + Vinorelbine 4 mg/kg | -0.1350 | 1.012544 | -2.2028907 | 1.932891 | -0.1333275 | 30 | 0.8948247 | 0.8948247 |
| Entinostat 4 mg/kg vs Entinostat 4 mg/kg + Topotecan 0.15 mg/kg | 2.5300 | 1.012544 | 0.4621093 | 4.597891 | 2.4986569 | 30 | 0.0181723 | 0.0441327 |
| Cyclophosphamide 50 mg/kg vs Entinostat 4 mg/kg + Cyclophosphamide 50 mg/kg | -0.1475 | 1.012544 | -2.2153907 | 1.920391 | -0.1456727 | 30 | 0.8851542 | 0.8948247 |
| Doxorubicin 2.5 mg/kg vs Entinostat 4 mg/kg + Doxorubicin 2.5 mg/kg | 0.9325 | 1.012544 | -1.1353907 | 3.000391 | 0.9209476 | 30 | 0.3644247 | 0.4765554 |
| Vinorelbine 4 mg/kg vs Entinostat 4 mg/kg + Vinorelbine 4 mg/kg | -0.9700 | 1.012544 | -3.0378907 | 1.097891 | -0.9579831 | 30 | 0.3457285 | 0.4765554 |
| Topotecan 0.15 mg/kg vs Entinostat 4 mg/kg + Topotecan 0.15 mg/kg | 1.2725 | 1.012544 | -0.7953907 | 3.340391 | 1.2567355 | 30 | 0.2185469 | 0.3377543 |

**Supplemental Table 3. Statistics for Tumor volume of CTG-1916**

| group | diff | se | lower | upper | t | df | pv | adjusted.p |
| --- | --- | --- | --- | --- | --- | --- | --- | --- |
| Control vs Entinostat 4 mg/kg | 241.5000 | 324.9245 | -422.084348 | 905.0843 | 0.7432496 | 30 | 0.4631118 | 0.5623501 |
| Control vs Cyclophosphamide 50 mg/kg | -183.5000 | 324.9245 | -847.084348 | 480.0843 | -0.5647466 | 30 | 0.5764450 | 0.6185272 |
| Control vs Doxorubicin 3 mg/kg | 395.6992 | 328.4253 | -275.034739 | 1066.4331 | 1.2048377 | 30 | 0.2376848 | 0.5451144 |
| Control vs Vinorelbine 4 mg/kg | 275.2500 | 324.9245 | -388.334348 | 938.8343 | 0.8471199 | 30 | 0.4036359 | 0.5451144 |
| Control vs Topotecan 0.15 mg/kg | 386.0000 | 324.9245 | -277.584348 | 1049.5843 | 1.1879683 | 30 | 0.2441663 | 0.5451144 |
| Control vs Entinostat 4 mg/kg + Cyclophosphamide | 314.0000 | 324.9245 | -349.584348 | 977.5843 | 0.9663784 | 30 | 0.3415812 | 0.5451144 |
| 50 mg/kg  Control vs Entinostat 4 mg/kg + Doxorubicin 3 mg/kg | 669.2500 | 324.9245 | 5.665652 | 1332.8343 | 2.0597093 | 30 | 0.0481950 | 0.5451144 |
| Control vs Entinostat 4 mg/kg + Vinorelbine 4 mg/kg | 542.7500 | 324.9245 | -120.834348 | 1206.3343 | 1.6703881 | 30 | 0.1052452 | 0.5451144 |
| Control vs Entinostat 4 mg/kg + Topotecan 0.15 mg/kg | 566.7500 | 324.9245 | -96.834348 | 1230.3343 | 1.7442514 | 30 | 0.0913547 | 0.5451144 |
| Entinostat 4 mg/kg vs Entinostat 4mg/kg + Cyclophosphamide 50 mg/kg | 72.5000 | 324.9245 | -591.084348 | 736.0843 | 0.2231288 | 30 | 0.8249476 | 0.8249476 |
| Entinostat 4 mg/kg vs Entinostat 4 mg/kg + Doxorubicin 3 mg/kg | 427.7500 | 324.9245 | -235.834348 | 1091.3343 | 1.3164597 | 30 | 0.1979894 | 0.5451144 |
| Entinostat 4 mg/kg vs Entinostat 4 mg/kg + Vinorelbine 4 mg/kg | 301.2500 | 324.9245 | -362.334348 | 964.8343 | 0.9271385 | 30 | 0.3612540 | 0.5451144 |
| Entinostat 4 mg/kg vs Entinostat 4 mg/kg + Topotecan 0.15 mg/kg | 325.2500 | 324.9245 | -338.334348 | 988.8343 | 1.0010018 | 30 | 0.3248320 | 0.5451144 |
| Cyclophosphamide 50 mg/kg vs Entinostat 4 mg/kg + Cyclophosphamide 50 mg/kg | 497.5000 | 324.9245 | -166.084348 | 1161.0843 | 1.5311249 | 30 | 0.1362166 | 0.5451144 |
| Doxorubicin 3 mg/kg vs Entinostat 4 mg/kg + Doxorubicin 3 mg/kg | 273.5508 | 328.4253 | -397.183105 | 944.2847 | 0.8329164 | 30 | 0.4114741 | 0.5451144 |
| Vinorelbine 4 mg/kg vs Entinostat 4 mg/kg + Vinorelbine 4 mg/kg | 267.5000 | 324.9245 | -396.084348 | 931.0843 | 0.8232682 | 30 | 0.4168522 | 0.5451144 |
| Topotecan 0.15 mg/kg vs Entinostat 4 mg/kg + Topotecan 0.15 mg/kg | 180.7500 | 324.9245 | -482.834348 | 844.3343 | 0.5562831 | 30 | 0.5821433 | 0.6185272 |

**Supplemental Table 4. Statistics for Body weight of CTG-1916**

| group | diff | se | lower | upper | t | df | pv | adjusted.p |
| --- | --- | --- | --- | --- | --- | --- | --- | --- |
| Control vs Entinostat 4 mg/kg | -0.59250 | 1.551362 | -3.7608033 | 2.5758033 | -0.3819225 | 30 | 0.7052109 | 0.7992390 |
| Control vs Cyclophosphamide 50 mg/kg | 0.06250 | 1.551362 | -3.1058033 | 3.2308033 | 0.0402872 | 30 | 0.9681311 | 0.9681311 |
| Control vs Doxorubicin 3 mg/kg | 3.57795 | 1.567615 | 0.3764537 | 6.7794472 | 2.2824167 | 30 | 0.0297241 | 0.1684364 |
| Control vs Vinorelbine 4 mg/kg | -2.35500 | 1.551362 | -5.5233033 | 0.8133033 | -1.5180212 | 30 | 0.1394779 | 0.3963361 |
| Control vs Topotecan 0.15 mg/kg | -2.35250 | 1.551362 | -5.5208033 | 0.8158033 | -1.5164097 | 30 | 0.1398833 | 0.3963361 |
| Control vs Entinostat 4 mg/kg + Cyclophosphamide | 0.69000 | 1.551362 | -2.4783033 | 3.8583033 | 0.4447705 | 30 | 0.6596780 | 0.7992390 |
| 50 mg/kg  Control vs Entinostat 4 mg/kg + Doxorubicin 3 mg/kg | 5.17750 | 1.551362 | 2.0091967 | 8.3458033 | 3.3373906 | 30 | 0.0022669 | 0.0192688 |
| Control vs Entinostat 4 mg/kg + Vinorelbine 4 mg/kg | -1.22750 | 1.551362 | -4.3958033 | 1.9408033 | -0.7912404 | 30 | 0.4350151 | 0.7992390 |
| Control vs Entinostat 4 mg/kg + Topotecan 0.15 mg/kg | 0.38500 | 1.551362 | -2.7833033 | 3.5533033 | 0.2481691 | 30 | 0.8056945 | 0.8560504 |
| Entinostat 4 mg/kg vs Entinostat 4mg/kg + Cyclophosphamide 50 mg/kg | 1.28250 | 1.551362 | -1.8858033 | 4.4508033 | 0.8266931 | 30 | 0.4149381 | 0.7992390 |
| Entinostat 4 mg/kg vs Entinostat 4 mg/kg + Doxorubicin 3 mg/kg | 5.77000 | 1.551362 | 2.6016967 | 8.9383033 | 3.7193131 | 30 | 0.0008207 | 0.0139516 |
| Entinostat 4 mg/kg vs Entinostat 4 mg/kg + Vinorelbine 4 mg/kg | -0.63500 | 1.551362 | -3.8033033 | 2.5333033 | -0.4093178 | 30 | 0.6852142 | 0.7992390 |
| Entinostat 4 mg/kg vs Entinostat 4 mg/kg + Topotecan 0.15 mg/kg | 0.97750 | 1.551362 | -2.1908033 | 4.1458033 | 0.6300916 | 30 | 0.5334049 | 0.7992390 |
| Cyclophosphamide 50 mg/kg vs Entinostat 4 mg/kg + Cyclophosphamide 50 mg/kg | 0.62750 | 1.551362 | -2.5408033 | 3.7958033 | 0.4044834 | 30 | 0.6887268 | 0.7992390 |
| Doxorubicin 3 mg/kg vs Entinostat 4 mg/kg + Doxorubicin 3 mg/kg | 1.59955 | 1.567615 | -1.6019472 | 4.8010463 | 1.0203715 | 30 | 0.3157108 | 0.7667262 |
| Vinorelbine 4 mg/kg vs Entinostat 4 mg/kg + Vinorelbine 4 mg/kg | 1.12750 | 1.551362 | -2.0408033 | 4.2958033 | 0.7267809 | 30 | 0.4729936 | 0.7992390 |
| Topotecan 0.15 mg/kg vs Entinostat 4 mg/kg + Topotecan 0.15 mg/kg | 2.73750 | 1.551362 | -0.4308033 | 5.9058033 | 1.7645788 | 30 | 0.0878152 | 0.3732146 |

**Supplemental Table 5. Statistics for Tumor Volume of CTG-2127**

| group | diff | se | lower | upper | t | df | pv | adjusted.p |
| --- | --- | --- | --- | --- | --- | --- | --- | --- |
| Control vs Entinostat 4 mg/kg | 481.66713 | 233.8853 | -6.209056 | 969.5433 | 2.059416 | 20 | 0.0527118 | 0.2404454 |
| Control vs Cyclophosphamide 50 mg/kg | 169.57445 | 232.8939 | -316.233724 | 655.3826 | 0.7281189 | 20 | 0.4749801 | 0.8074662 |
| Control vs Doxorubicin 3 mg/kg | 10.80389 | 233.9007 | -477.104434 | 498.7122 | 0.0461901 | 20 | 0.9636169 | 0.9636169 |
| Control vs Vinorelbine 4 mg/kg | 111.81837 | 232.8939 | -373.98981 | 597.6265 | 0.4801258 | 20 | 0.6363474 | 0.9557266 |
| Control vs Topotecan 0.15 mg/kg | 72.46068 | 233.7093 | -415.048385 | 559.9697 | 0.3100462 | 20 | 0.7597334 | 0.9557266 |
| Control vs Entinostat 4 mg/kg + Cyclophosphamide 50 mg/kg | 424.04364 | 233.8853 | -63.832554 | 911.9198 | 1.813041 | 20 | 0.0848631 | 0.2404454 |
| Control vs Entinostat 4 mg/kg + Doxorubicin 3 mg/kg | 450.05458 | 233.8853 | -37.821615 | 937.9308 | 1.9242533 | 20 | 0.0686698 | 0.2404454 |
| Control vs Entinostat 4 mg/kg + Vinorelbine 4 mg/kg | 511.74797 | 233.8853 | 23.871784 | 999.6242 | 2.1880296 | 20 | 0.0407085 | 0.2404454 |
| Control vs Entinostat 4 mg/kg + Topotecan 0.15 mg/kg | 438.13815 | 233.8853 | -49.738043 | 926.0143 | 1.8733035 | 20 | 0.0757161 | 0.2404454 |
| Entinostat 4 mg/kg vs Entinostat 4mg/kg + Cyclophosphamide 50 mg/kg | -57.6235 | 235.195 | -548.231715 | 432.9847 | -0.2450031 | 20 | 0.8089504 | 0.9557266 |
| Entinostat 4 mg/kg vs Entinostat 4 mg/kg + Doxorubicin 3 mg/kg | -31.61256 | 235.195 | -522.220776 | 458.9957 | -0.13441 | 20 | 0.8944218 | 0.9557266 |
| Entinostat 4 mg/kg vs Entinostat 4 mg/kg + Vinorelbine 4 mg/kg | 30.08084 | 235.195 | -460.527377 | 520.6891 | 0.1278974 | 20 | 0.8995074 | 0.9557266 |
| Entinostat 4 mg/kg vs Entinostat 4 mg/kg + Topotecan 0.15 mg/kg | -43.52899 | 235.195 | -534.137204 | 447.0792 | -0.1850761 | 20 | 0.8550333 | 0.9557266 |
| Cyclophosphamide 50 mg/kg vs Entinostat 4 mg/kg + Cyclophosphamide 50 mg/kg | 254.46918 | 234.2092 | -234.082583 | 743.021 | 1.0865039 | 20 | 0.2901782 | 0.5481143 |
| Doxorubicin 3 mg/kg vs Entinostat 4 mg/kg + Doxorubicin 3 mg/kg | 439.25069 | 235.2103 | -51.38948 | 929.8909 | 1.8674804 | 20 | 0.0765606 | 0.2404454 |
| Vinorelbine 4 mg/kg vs Entinostat 4 mg/kg + Vinorelbine 4 mg/kg | 399.92961 | 234.2092 | -88.622158 | 888.4814 | 1.7075745 | 20 | 0.1031929 | 0.2506114 |
| Topotecan 0.15 mg/kg vs Entinostat 4 mg/kg + Topotecan 0.15 mg/kg | 365.67747 | 235.02 | -124.565664 | 855.9206 | 1.5559419 | 20 | 0.1354049 | 0.2877354 |

**Supplemental Table 6. Statistics for Body Weight of CTG-2127**

| group | diff | se | lower | upper | t | df | pv | adjusted.p |
| --- | --- | --- | --- | --- | --- | --- | --- | --- |
| Control vs Entinostat 4 mg/kg | -1.3628052 | 1.229964 | -3.9284651 | 1.2028548 | -1.1080041 | 20 | 0.2810076 | 0.577151 |
| Control vs Cyclophosphamide 50 mg/kg | -1.7361091 | 1.22947 | -4.3007394 | 0.8285213 | -1.4120788 | 20 | 0.1732976 | 0.533074 |
| Control vs Doxorubicin 3 mg/kg | -1.4456073 | 1.23005 | -4.0114473 | 1.1202326 | -1.1752424 | 20 | 0.2536999 | 0.577151 |
| Control vs Vinorelbine 4 mg/kg | -1.8953643 | 1.22947 | -4.4599947 | 0.669266 | -1.5416104 | 20 | 0.1388427 | 0.533074 |
| Control vs Topotecan 0.15 mg/kg | -1.9951456 | 1.230096 | -4.5610803 | 0.5707892 | -1.6219433 | 20 | 0.1204745 | 0.533074 |
| Control vs Entinostat 4 mg/kg + Cyclophosphamide 50 mg/kg | -0.6065281 | 1.229964 | -3.172188 | 1.9591319 | -0.4931267 | 20 | 0.627294 | 0.888667 |
| Control vs Entinostat 4 mg/kg + Doxorubicin 3 mg/kg | -1.2933565 | 1.229964 | -3.8590165 | 1.2723034 | -1.0515401 | 20 | 0.3055507 | 0.577151 |
| Control vs Entinostat 4 mg/kg + Vinorelbine 4 mg/kg | -1.6759998 | 1.229964 | -4.2416598 | 0.8896601 | -1.3626414 | 20 | 0.1881436 | 0.533074 |
| Control vs Entinostat 4 mg/kg + Topotecan 0.15 mg/kg | 0.4931412 | 1.229964 | -2.0725188 | 3.0588011 | 0.4009395 | 20 | 0.6927158 | 0.905859 |
| Entinostat 4 mg/kg vs Entinostat 4mg/kg + Cyclophosphamide 50 mg/kg | 0.7562771 | 1.230531 | -1.8105649 | 3.3231191 | 0.6145943 | 20 | 0.5457519 | 0.843435 |
| Entinostat 4 mg/kg vs Entinostat 4 mg/kg + Doxorubicin 3 mg/kg | 0.0694487 | 1.230531 | -2.4973934 | 2.6362907 | 0.056438 | 20 | 0.9555529 | 0.955553 |
| Entinostat 4 mg/kg vs Entinostat 4 mg/kg + Vinorelbine 4 mg/kg | -0.3131947 | 1.230531 | -2.8800367 | 2.2536474 | -0.25452 | 20 | 0.8016925 | 0.955553 |
| Entinostat 4 mg/kg vs Entinostat 4 mg/kg + Topotecan 0.15 mg/kg | 1.8559464 | 1.230531 | -0.7108957 | 4.4227884 | 1.5082488 | 20 | 0.1471261 | 0.533074 |
| Cyclophosphamide 50 mg/kg vs Entinostat 4 mg/kg + Cyclophosphamide 50 mg/kg | 1.129581 | 1.230037 | -1.4362319 | 3.6953939 | 0.9183307 | 20 | 0.3693887 | 0.627961 |
| Doxorubicin 3 mg/kg vs Entinostat 4 mg/kg + Doxorubicin 3 mg/kg | 0.1522508 | 1.230617 | -2.4147711 | 2.7192727 | 0.1237191 | 20 | 0.9027726 | 0.955553 |
| Vinorelbine 4 mg/kg vs Entinostat 4 mg/kg + Vinorelbine 4 mg/kg | 0.2193645 | 1.230037 | -2.3464484 | 2.7851774 | 0.1783397 | 20 | 0.8602503 | 0.955553 |
| Topotecan 0.15 mg/kg vs Entinostat 4 mg/kg + Topotecan 0.15 mg/kg | 2.4882868 | 1.230662 | -0.0788299 | 5.0554035 | 2.0219086 | 20 | 0.0567686 | 0.533074 |

**Supplemental Table 7. Statistics for Tumor volume of CTG-1213**

| group | diff | se | lower | upper | t | df | pv | adjusted.p |
| --- | --- | --- | --- | --- | --- | --- | --- | --- |
| Control vs Entinostat 4 mg/kg | 486.2566 | 195.3740 | 87.24968 | 885.2635 | 2.4888502 | 30 | 0.0185908 | 0.0526741 |
| Control vs Cyclophosphamide 50 mg/kg | 451.7566 | 195.3740 | 52.74968 | 850.7635 | 2.3122658 | 30 | 0.0278080 | 0.0675336 |
| Control vs Doxorubicin 2.5 mg/kg | 417.5066 | 195.3740 | 18.49968 | 816.5135 | 2.1369610 | 30 | 0.0408721 | 0.0772029 |
| Control vs Vinorelbine 4 mg/kg | 428.2566 | 195.3740 | 29.24968 | 827.2635 | 2.1919837 | 30 | 0.0362778 | 0.0770902 |
| Control vs Topotecan 0.15 mg/kg | 542.5066 | 195.3740 | 143.49968 | 941.5135 | 2.7767596 | 30 | 0.0093699 | 0.0318576 |
| Control vs Entinostat 4 mg/kg + Cyclophosphamide | 766.7566 | 195.3740 | 367.74968 | 1165.7635 | 3.9245582 | 30 | 0.0004696 | 0.0079836 |
| 50 mg/kg  Control vs Entinostat 4 mg/kg + Doxorubicin 2.5 mg/kg | 637.0066 | 195.3740 | 237.99968 | 1036.0135 | 3.2604473 | 30 | 0.0027700 | 0.0123728 |
| Control vs Entinostat 4 mg/kg + Vinorelbine 4 mg/kg | 633.2566 | 195.3740 | 234.24968 | 1032.2635 | 3.2412534 | 30 | 0.0029112 | 0.0123728 |
| Control vs Entinostat 4 mg/kg + Topotecan 0.15 mg/kg | 657.5066 | 195.3740 | 258.49968 | 1056.5135 | 3.3653743 | 30 | 0.0021067 | 0.0123728 |
| Entinostat 4 mg/kg vs Entinostat 4mg/kg + Cyclophosphamide 50 mg/kg | 280.5000 | 190.6355 | -108.82962 | 669.8296 | 1.4713944 | 30 | 0.1515974 | 0.2342869 |
| Entinostat 4 mg/kg vs Entinostat 4 mg/kg + Doxorubicin 2.5 mg/kg | 150.7500 | 190.6355 | -238.57962 | 540.0796 | 0.7907761 | 30 | 0.4352819 | 0.4745930 |
| Entinostat 4 mg/kg vs Entinostat 4 mg/kg + Vinorelbine 4 mg/kg | 147.0000 | 190.6355 | -242.32962 | 536.3296 | 0.7711051 | 30 | 0.4466758 | 0.4745930 |
| Entinostat 4 mg/kg vs Entinostat 4 mg/kg + Topotecan 0.15 mg/kg | 171.2500 | 190.6355 | -218.07962 | 560.5796 | 0.8983112 | 30 | 0.3761732 | 0.4567817 |
| Cyclophosphamide 50 mg/kg vs Entinostat 4 mg/kg + Cyclophosphamide 50 mg/kg | 315.0000 | 190.6355 | -74.32962 | 704.3296 | 1.6523680 | 30 | 0.1088893 | 0.1851119 |
| Doxorubicin 2.5 mg/kg vs Entinostat 4 mg/kg + Doxorubicin 2.5 mg/kg | 219.5000 | 190.6355 | -169.82962 | 608.8296 | 1.1514120 | 30 | 0.2586570 | 0.3664307 |
| Vinorelbine 4 mg/kg vs Entinostat 4 mg/kg + Vinorelbine 4 mg/kg | 205.0000 | 190.6355 | -184.32962 | 594.3296 | 1.0753506 | 30 | 0.2907905 | 0.3802646 |
| Topotecan 0.15 mg/kg vs Entinostat 4 mg/kg + Topotecan 0.15 mg/kg | 115.0000 | 190.6355 | -274.32962 | 504.3296 | 0.6032455 | 30 | 0.5508794 | 0.5508794 |

**Supplemental Table 8. Statistics for Body weight of CTG-1213**

| group | diff | se | lower | upper | t | df | pv | adjusted.p |
| --- | --- | --- | --- | --- | --- | --- | --- | --- |
| Control vs Entinostat 4 mg/kg | -1.2394981 | 1.155354 | -3.5990455 | 1.120049 | -1.0728298 | 30 | 0.2919019 | 0.6956877 |
| Control vs Cyclophosphamide 50 mg/kg | -0.9694981 | 1.155354 | -3.3290455 | 1.390049 | -0.8391352 | 30 | 0.4080306 | 0.6956877 |
| Control vs Doxorubicin 2.5 mg/kg | 0.7305019 | 1.155354 | -1.6290455 | 3.090049 | 0.6322755 | 30 | 0.5319965 | 0.6956877 |
| Control vs Vinorelbine 4 mg/kg | -1.1594981 | 1.155354 | -3.5190455 | 1.200049 | -1.0035870 | 30 | 0.3236043 | 0.6956877 |
| Control vs Topotecan 0.15 mg/kg | -0.9119981 | 1.155354 | -3.2715455 | 1.447549 | -0.7893669 | 30 | 0.4360922 | 0.6956877 |
| Control vs Entinostat 4 mg/kg + Cyclophosphamide | 0.5830019 | 1.155354 | -1.7765455 | 2.942549 | 0.5046090 | 30 | 0.6175201 | 0.7342729 |
| 50 mg/kg  Control vs Entinostat 4 mg/kg + Doxorubicin 2.5 mg/kg | -0.2819981 | 1.155354 | -2.6415455 | 2.077549 | -0.2440794 | 30 | 0.8088309 | 0.8088309 |
| Control vs Entinostat 4 mg/kg + Vinorelbine 4 mg/kg | -0.3819981 | 1.155354 | -2.7415455 | 1.977549 | -0.3306330 | 30 | 0.7432199 | 0.7896711 |
| Control vs Entinostat 4 mg/kg + Topotecan 0.15 mg/kg | 0.5330019 | 1.155354 | -1.8265455 | 2.892549 | 0.4613322 | 30 | 0.6478879 | 0.7342729 |
| Entinostat 4 mg/kg vs Entinostat 4mg/kg + Cyclophosphamide 50 mg/kg | 1.8225000 | 1.138706 | -0.5030473 | 4.148047 | 1.6005013 | 30 | 0.1199670 | 0.6956877 |
| Entinostat 4 mg/kg vs Entinostat 4 mg/kg + Doxorubicin 2.5 mg/kg | 0.9575000 | 1.138706 | -1.3680473 | 3.283047 | 0.8408669 | 30 | 0.4070749 | 0.6956877 |
| Entinostat 4 mg/kg vs Entinostat 4 mg/kg + Vinorelbine 4 mg/kg | 0.8575000 | 1.138706 | -1.4680473 | 3.183047 | 0.7530479 | 30 | 0.4572903 | 0.6956877 |
| Entinostat 4 mg/kg vs Entinostat 4 mg/kg + Topotecan 0.15 mg/kg | 1.7725000 | 1.138706 | -0.5530473 | 4.098047 | 1.5565918 | 30 | 0.1300553 | 0.6956877 |
| Cyclophosphamide 50 mg/kg vs Entinostat 4 mg/kg + Cyclophosphamide 50 mg/kg | 1.5525000 | 1.138706 | -0.7730473 | 3.878047 | 1.3633900 | 30 | 0.1829045 | 0.6956877 |
| Doxorubicin 2.5 mg/kg vs Entinostat 4 mg/kg + Doxorubicin 2.5 mg/kg | -1.0125000 | 1.138706 | -3.3380473 | 1.313047 | -0.8891674 | 30 | 0.3809878 | 0.6956877 |
| Vinorelbine 4 mg/kg vs Entinostat 4 mg/kg + Vinorelbine 4 mg/kg | 0.7775000 | 1.138706 | -1.5480473 | 3.103047 | 0.6827927 | 30 | 0.4999769 | 0.6956877 |
| Topotecan 0.15 mg/kg vs Entinostat 4 mg/kg + Topotecan 0.15 mg/kg | 1.4450000 | 1.138706 | -0.8805473 | 3.770547 | 1.2689846 | 30 | 0.2142042 | 0.6956877 |
